# Diphosphine-Substituted Rhodium Carbonyl Clusters: Synthesis and Structural and Spectroscopic Characterization of the Heteroleptic Rh_4_(CO)_8+2n_(L)_2−n_ (n = 0, 1) and {Rh_4_(CO)_10_L}_2_ Monomeric and Dimeric Species

**DOI:** 10.3390/molecules31010193

**Published:** 2026-01-05

**Authors:** Giorgia Scorzoni, Guido Bussoli, Cristiana Cesari, Maria Carmela Iapalucci, Stefano Zacchini, Cristina Femoni

**Affiliations:** Department of Industrial Chemistry “Toso Montanari”, University of Bologna, Via Gobetti 85, 40129 Bologna, Italy; giorgia.scorzoni3@unibo.it (G.S.); bussoliguido@gmail.com (G.B.); cristiana.cesari2@unibo.it (C.C.); stefano.zacchini@unibo.it (S.Z.)

**Keywords:** rhodium, carbonyl clusters, diphosphine ligands, X-ray diffraction, NMR analysis

## Abstract

Tetranuclear rhodium carbonyl clusters are vital catalytic precursors; yet derivatives featuring bidentate phosphines are less common, due to the propensity for cluster fragmentation during synthesis. This study reports the successful isolation of five new heteroleptic species by reacting Rh_4_(CO)_12_ with various bidentate diphosphines under homogeneous conditions and at room temperature, namely the mono-substituted Rh_4_(CO)_10_(dppe) (**1**) and Rh_4_(CO)_10_(dppb) (**3**), the rare bis-substituted derivative Rh_4_(CO)_8_(dppe)_2_ (**2**), and the two unique dimeric assemblies {Rh_4_(CO)_10_(dpp-hexane)}_2_ (**4**) and {Rh_4_(CO)_10_(*trans*-dppe)}_2_ (**5**). The tetrahedral Rh_4_ core of the cluster precursor was preserved in all cases. The new compounds were characterized via infrared (IR) spectroscopy and single-crystal X-ray diffraction (SC-XRD). Furthermore, variable-temperature (VT) ^31^P{^1^H} NMR spectroscopy elucidated the dynamic behavior of the phosphorus atoms. This work reports a robust methodology for accessing stable, low-nuclearity rhodium phosphine clusters with tunable properties.

## 1. Introduction

Metal carbonyl clusters (MCCs) represent a distinctive class of organometallic compounds defined by the presence of metal–metal bonds and in which carbonyls act not only as simple ligands but also as stabilizing and structure-directing agents [1,2], giving rise to compact and highly symmetric structures ranging from simple dimers to large, multi-metal frameworks [3]. The fluxional behavior of the carbonyl ligands in solution, bridging or terminal, also adds to their dynamic chemistry [4,5]. The bonding in MCCs and their correlation with the geometry of their metal skeleton are often described using the Wade–Mingos electron-counting rules [6] and delocalized molecular orbital models. More specifically, a bilateral correspondence between the geometry of a given MCC and its electron counting has been consistently observed. However, as the cluster nuclearity (number of metals) increases, such correspondence may fail and clusters can exhibit remarkable electronic [7] and magnetic [8] features, as well as interesting catalytic properties [9]. Moreover, when MCCs reach high metal nuclearity (from about twelve metal atoms onwards), they are often referred to as “atomically precise nanoclusters” [10], because they are identified at a molecular level despite possessing nanometric dimensions. Rhodium, as well as platinum, is among those metals that may form carbonyl species with a nanometric size under suitable conditions [11,12,13], thanks to its high metal–metal and metal–CO bond energy [14].

On the other hand, low-nuclearity carbonyl species also possess interesting properties; for instance, they often serve as homogeneous catalysts or catalyst precursors for hydroformylation [15] and hydrogenation [16] reactions. Moreover, their multi-metal active sites allow cooperative mechanisms resembling those of heterogeneous catalysts, but with the tunability of molecular systems. Among MCCs, rhodium species have been studied for their catalytic abilities [17] and, for instance, the activity of Rh_4_(CO)_12_ and Rh_6_(CO)_16_ has been found comparable to that of mononuclear rhodium complexes in hydroformylation reactions [18,19].

Despite their strong coordination with the metal core, under suitable conditions, carbonyl groups can be partially replaced by other ligands [20], allowing for tuning the MCCs’ properties and enhancing their catalytic properties. In the literature, phosphine-substituted rhodium carbonyl clusters are synthesized from the neutral Rh_6_(CO)_16_ by partially replacing the carbonyl ligands with either mono- [21] or diphosphines [22], yielding species like Rh_6_(CO)_10_L_6_ (L = mono-phosphine ligand), Rh_6_(CO)_12_L_2_ (L = 1,4-bis(diphenylphosphino)butane), and Rh_6_(CO)_16−x_L_x_ (L = 1,2-bis(diphenylphosphino)ethane, x = 1, 3). Notably, in the latter clusters, the diphosphine is always coordinated with just one rhodium atom, acting as a monodentate ligand. A key example of a heterogeneous catalyst made of a phosphine-substituted rhodium cluster is reported by Iwatate K. et al. [23]. They described how Rh_6_(CO)_16_ could bind a modified polystyrene surface via a simple ligand substitution reaction in which phosphines attached to the polymer replace carbonyls of the cluster. In addition, the catalyst obtained can be recycled and refreshed in a carbon monoxide atmosphere, once used. This kind of recycling is crucial considering that rhodium is a costly transition metal. Carbonyl substitution reactions with phosphine ligands are also reported on Rh_4_(CO)_12_ [24,25]. In general terms, reactions of Rh_4_(CO)_12_ with P-donor ligands (PR_3_, phosphites, etc.), carried out in mild conditions, typically yield heteroleptic clusters of the type Rh_4_(CO)_12−x_L_x_ (x = 2–4 are commonly observed), e.g., Rh_4_(CO)_10_L_2_, Rh_4_(CO)_9_L_3_, and Rh_4_(CO)_8_L_4_, with the Rh_4_ metal skeleton usually retained.

In the case of diphosphine ligands, while Rh_6_(CO)_16−2x_L_x_ (L = 1, 2) clusters are widely known [26,27,28], there is a lack of literature on diphosphine-substituted clusters synthesized from Rh_4_(CO)_12_, with the exception of Rh_4_(CO)_8_L_2_ (L = bis(diphenylphosphino)methane) [29]. One possible reason could be that, under certain conditions (especially in the presence of CO, with certain bulky/very nucleophilic phosphines), cluster breakdown can occur and give smaller rhodium species (e.g., dinuclear Rh–Rh bridged compounds) or mixed metal fragments, so substitution does not always preserve the tetranuclear core [30].

Therefore, we decided to fill this gap and explore the possibility of preparing new heteroleptic carbonyl clusters by reacting Rh_4_(CO)_12_ with different diphosphine species. Reactions were carried out under a nitrogen atmosphere, at room temperature, and in homogeneous conditions. We successfully isolated and identified five new species, namely Rh_4_(CO)_10_(dppe) (**1**), its parent bis-substituted derivative Rh_4_(CO)_8_(dppe)_2_ (**2**), Rh_4_(CO)_10_(dppb) (**3**), and the dimeric {Rh_4_(CO)_10_(dpp-hexane)}_2_ (**4**) and {Rh_4_(CO)_10_(*trans*-dppe)}_2_ (**5**) clusters [dppe = 1,2-bis(diphenylphosphino)ethane; dppb = 1,4-bis(diphenylphosphino)butane; *trans*-dppe = *trans*-1,2-bis(diphenylphosphino)ethylene; dpp-hexane = 1,6-bis(diphenylphosphino)hexane]. Every species has been isolated and characterized via single-crystal X-ray diffraction (SC-XRD) and infrared (IR) spectroscopy. To complete their characterization and investigate the behavior of the phosphorus atoms in solution, ^31^P{^1^H} NMR studies at variable temperature (VT) were performed on each cluster. The crystal structure of Rh_4_(CO)_10_(dppb) evidenced serious disorder issues that prevented the obtainment of good crystallographic data; nevertheless, we still could obtain a structural model perfectly consistent with the NMR analysis.

## 2. Results and Discussion

**Scheme 1:**
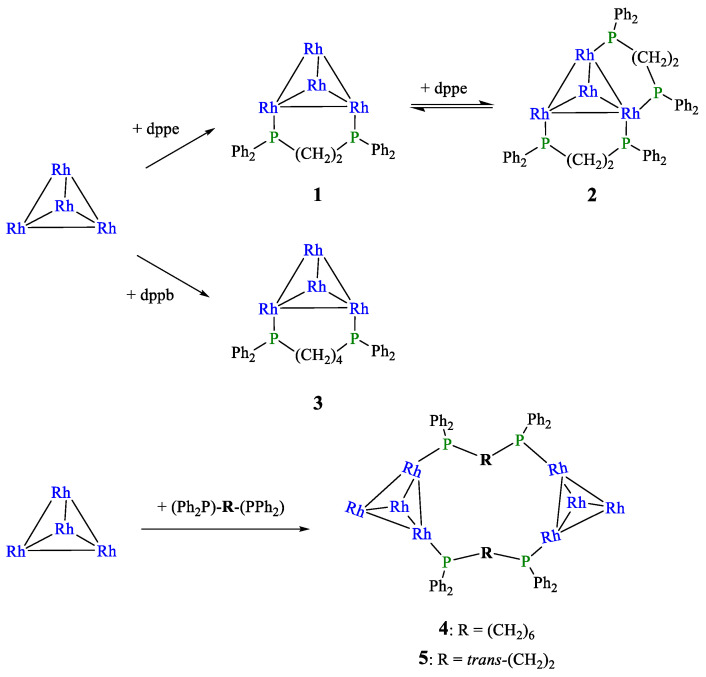
Summary of the synthesis of mono-, bi-chelated, and dimeric phosphine-substituted rhodium carbonyl clusters. Carbonyl ligands are omitted for clarity.

The reactivity of Rh_4_(CO)_12_ with various diphosphines has been investigated at room temperature, under an inert atmosphere and in homogeneous conditions, to have all reactants and products in solution. A nitrogen atmosphere was employed to facilitate the carbonyl substitution and minimize possible cluster fragmentation, as reported in the literature [21]. The ligands—dppe, dppb, dpp-hexane, and *trans*-dppe—were selected to evaluate the influence of distance and geometrical constraints between the phosphorus centers on their coordination ability towards the cluster. Indeed, when reacting Rh_4_(CO)_12_ with one equivalent of the phosphine ligand, in the case of dppe and dppb, the diphosphine replaced two carbonyls within the same Rh_4_(CO)_10_ tetrahedron, acting as an intramolecular bidentate ligand and producing monochelated species (compounds **1** and **3** in Scheme 1). Conversely, in the case of dpp-hexane, which possesses the longer aliphatic carbon chain, the diphosphine linked two different tetrahedra, acting as an intermolecular bidentate ligand, thus forming a dimeric species with a general formula {Rh_4_(CO)_10_(Ph_2_P-R-PPh_2_)}_2_ (compound **4**). The same occurred when *trans*-dppe was employed (compound **5**). Finally, only in the case of dppe, by adding two equivalents with respect to the starting Rh_4_(CO)_12_, it was also possible to obtain the corresponding bis-chelated cluster (compound **2**).

As a general procedure, the diphosphines were added at room temperature in powder form to a CH_2_Cl_2_ solution of the pre-formed Rh_4_(CO)_12_. In the literature [22,27], it is reported that the main positive effect achieved by conducting the reactions in heterogeneous conditions in non-polar solvents (such as hexane, benzene, chloroform, etc.) is the separation of the products through precipitation. However, as we discovered in our studies, the reactions are not as fast as when carried out in homogeneous conditions. Moreover, in non-polar solvents, the presence of the precursor after a 24 h reaction is still detectable by IR spectroscopy. Therefore, we carried out all reactions using CH_2_Cl_2_ as a solvent, which allowed us to solubilize both reactants and products, and we monitored their evolution path via IR spectroscopy. At the end of each reaction, the crude mixture was dried under a vacuum, and the product was extracted with the appropriate solvent and subjected to crystallization, performed by using the double-layer technique. To obtain crystals suitable for structural analysis, the best solvent combinations were selected through trial and error. For compounds **3**, **4**, and **5**, the solid product was extracted in toluene and layered with hexane, while the toluene extraction of **1** was layered with pentane. Better-quality crystals of **5** were obtained by re-dissolving the product in tetrahydrofuran (THF) and layering the solution with hexane. In the case of **2**, the solid product was washed with toluene and extracted in THF, and then the solution was combined with hexane.

The IR spectra of products **1**, **3**, **4**, and **5** registered in the CH_2_Cl_2_ solution, irrespective of the employed diphosphine ligand, displayed ν_CO_ bands in the ranges of 2068–2015 and 1845–1805 cm^−1^, corresponding to the stretching of terminal and edge-bridging carbonyl groups, respectively. If compared with the ν_CO_ values of the starting Rh_4_(CO)_12_ cluster (2072, 2043, and 1885 cm^−1^), these signals are congruent with the displacement of two carbonyl ligands out of the twelve coordinated to the parent compound. In fact, a ν_CO_ downshift ongoing from the homoleptic to the heteroleptic cluster is expected owing to the weaker Lewis acidity of the phosphine ligand, which in turn causes stronger overall π back-bonding between the rhodium atoms and the remaining carbonyls. A further decrease of ν_CO_ was observed in the unique case of **2**, when the second equivalent of dppe was added and two more carbonyl ligands were replaced, signaling the formation of the dichelated species (ν_CO_ = 2010–1982 and 1805–1783 cm^−1^).

In all cases, the reaction yields were rather high, except for compound **2**, as a significant amount of the monochelated species **1** was still residual. All the new mono-, dichelated, and dimeric species have been characterized via IR, ^31^P{^1^H} NMR spectroscopy, and SC-XRD. In the case of Rh_4_(CO)_10_(dppb), the low quality of the crystals prevented us from having a complete and convergent refinement of the structural parameters. Moreover, a heavy disorder issue in the solid state was present. However, we were still able to obtain a satisfactory structural model, which was in complete agreement with the NMR analysis. A further electrospray ionization mass-spectrometry (ESI-MS) characterization was tried for all the species herein reported, but heavy fragmentation was observed in all cases; therefore, no significant results were achieved.

**Figure 1:**
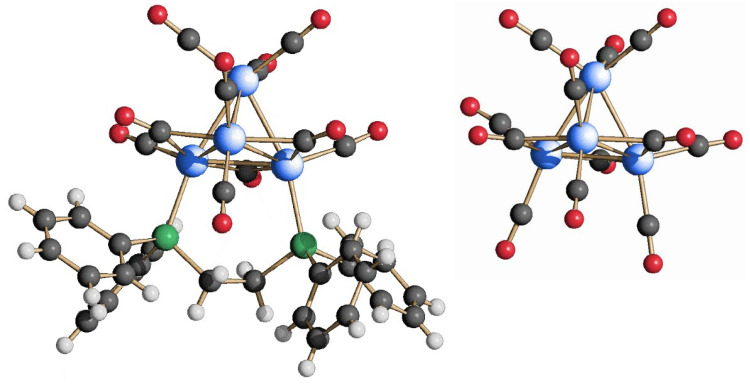
Molecular structure of Rh_4_(CO)_10_(dppe) (**1**) (**left**) and its comparison with that of Rh_4_(CO)_12_ (**right**) (blue, Rh; green, P; gray, C; red, O; white, H).

The heteroleptic cluster Rh_4_(CO)_10_(dppe) (**1**) was obtained by adding one equivalent of dppe to a CH_2_Cl_2_ solution of the Rh_4_(CO)_12_ precursor. By monitoring the reaction mixture via IR spectroscopy, it was possible to detect a clear downshift of the ν_CO_ stretching values after a few hours, indicating the substitution of two carbonyl ligands by the diphosphine. The product was extracted in toluene and crystallized by slowly adding a layer of pentane on top of the solution. The molecular structure of **1**, illustrated in Figure 1 (left), retains the original tetrahedral metal core of Rh_4_(CO)_12_ and its unbalanced carbonyl ligand distribution with respect to the metal centers. More specifically, in Rh_4_(CO)_12_, one rhodium atom differs from the others, being coordinated to three terminal carbonyl groups, whereas each of the remaining three rhodium atoms bears two terminal- and two edge-bridging carbonyls. As a result, despite the tetrahedral symmetry of the metal framework, the overall structure of Rh_4_(CO)_12_ (Figure 1, right) could be seen as a trigonal-based pyramid, the unique rhodium atom being its vertex and the other three representing the base. In compound **1**, the same ligand distribution as Rh_4_(CO)_12_ is retained except for two terminal carbonyl groups coordinated to the triangular base, which have been replaced by the phosphorus atoms of the bidentate dppe ligand.

**Figure 2:**
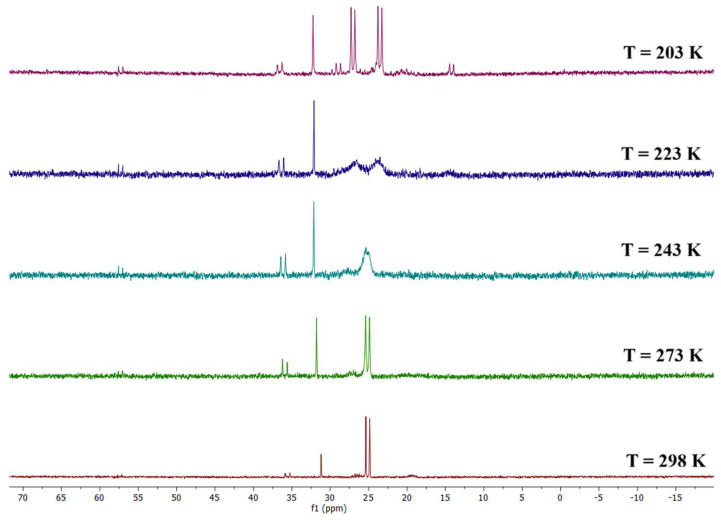
VT ^31^P{^1^H} NMR spectra of the species Rh_4_(CO)_10_(dppe) (**1**) in CD_2_Cl_2_ solution.

Despite the uneven ligand distribution of **1**, the overall cluster structure actually appears to be fairly symmetrical. As a matter of fact, a mirror plane going through the pyramid vertex and crossing the midpoint of the pyramid base’s edge can be identified, with only the -CH_2_CH_2_- spacer being the symmetry-disrupting element in the solid state (Figure S11, inset). Therefore, it was reasonable to assume that, in solution, the two phosphorus atoms could be chemically equivalent. This hypothesis was confirmed by the VT ^31^P{^1^H} NMR analysis, reported in Figure 2. In particular, the spectrum recorded at room temperature (Figure S11) presented only one doublet at δ_P_ = 25.11 ppm due to the Rh-P coupling (*J*_Rh-P_ = 124.1 Hz). However, as the temperature decreased, this signal broadened until the two phosphorus atoms coalesced at around T = 243 K; subsequently, their two related separate doublets clearly appeared at T = 203 K (δ: 27.04 (d, *J*_Rh-P_ = 121.8 Hz), 23.53 (d, *J*_Rh-P_ = 124.0 Hz) ppm). This phenomenon could be explained by the dynamic temperature-dependent behavior of the -CH_2_-CH_2_- chain between the phosphorus atoms, which is inhibited at a low temperature, thus breaking the symmetry of the molecule and meaning the phosphorus atoms are no longer chemically equivalent. A similar behavior had been observed for Rh_6_(CO)_14_(dppe) [27].

**Figure 3:**
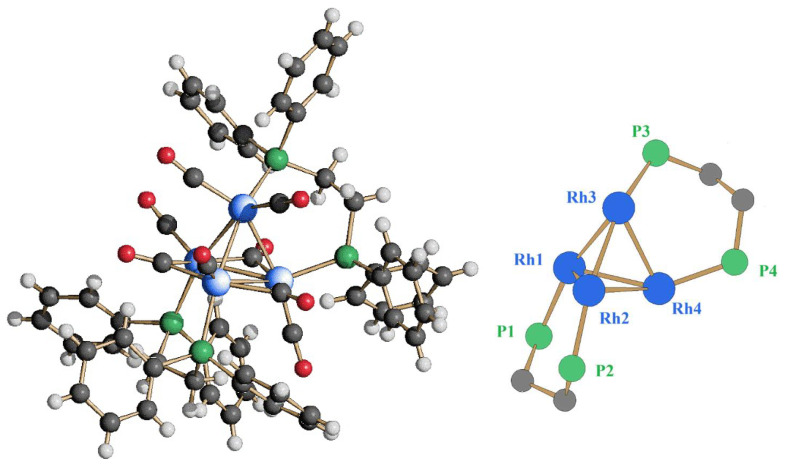
Molecular structure of Rh_4_(CO)_8_(dppe)_2_ (**2**) (**left**) and its coordination skeleton (**right**) (blue, Rh; green, P; gray, C; red, O; white, H).

When two equivalents of dppe were added to Rh_4_(CO)_12_, a further downshift of the ν_CO_ stretching values with respect to that observed for **1** was registered, indicating the formation of a new, more substituted dichelated product. By layering hexane on the THF extraction, crystals suitable for the SC-XRD analysis were obtained, allowing the identification of the new Rh_4_(CO)_8_(dppe)_2_ (**2**) cluster. Its molecular structure is reported in Figure 3. The second diphosphine is coordinated to the two rhodium atoms that were still phosphorus-free in compound **1**, acting too as a bidentate ligand. The end result is the replacement of a terminal carbonyl group on each rhodium atom with respect to the initial Rh_4_(CO)_12_. The residual eight carbonyls are divided into five terminally coordinated, one for each rhodium but with the vertex one bearing two, and three edge-bridging along the base. Unlike **1**, no symmetry could be envisaged for cluster **2**.

Possibly due to the steric encumbrance of the two diphosphines, the bis-chelated heteroleptic cluster was not as stable as the monochelated one, with **2** having the tendency to go back to **1**. The addition of an excess of dppe to shift this equilibrium in favor of **2** resulted in cluster degradation. Conversely, even a third diphosphine substitution was reported to occur through the addition of an excess of dppe ligand to Rh_6_(CO)_16_, yielding Rh_6_(CO)_13_(dppe)_3_ [22]; notably, however, the dppe here acted as monodentate ligands even when only one was coordinated to the parent cluster. It is possible to assume that also, in our case, the dppe would behave in the same way, preventing us from obtaining compound **2** in high yields.

**Figure 4:**
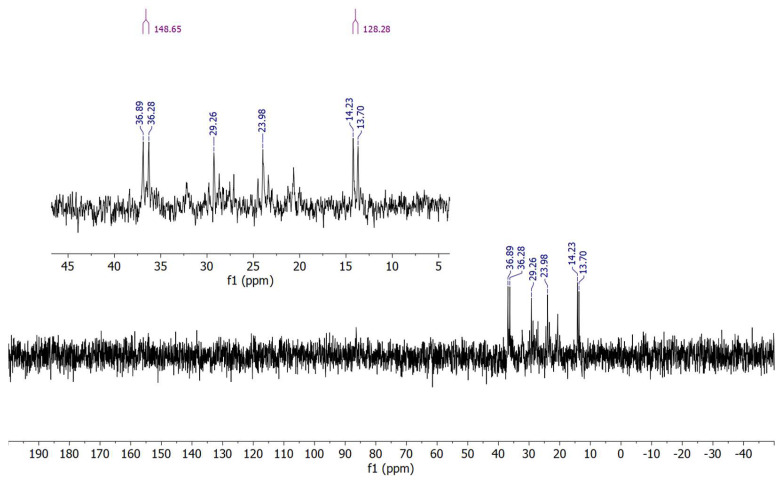
^31^P{^1^H} NMR spectrum of Rh_4_(CO)_8_(dppe)_2_ (**2**) in CD_2_Cl_2_ solution at T = 193 K.

Despite the difficulties in isolating cluster **2**, we were able to perform a VT ^31^P{^1^H} NMR analysis on a few obtained crystals, which were hand-picked to be sure of handling a pure sample. Judging from the crystal structure, the four phosphorus atoms in **2** are not chemically equivalent, so four separated signals were expected. At room temperature, the spectrum showed intense background noise due to the low concentration of the sample (Figure S14); moreover, the quality of the spectrum was worsened by the dynamic behavior of the ligands. Then, as the temperature decreased, the dynamic processes slowed down and a clearer spectrum was obtained at T = 193 K (Figure 4), indeed showing four separated signals, one for each phosphorus atom, thus confirming the initial hypothesis. Nonetheless, the spectrum presented two doublets and two triplets. This finding could be explained by analyzing the relative spatial coordination of the phosphorus atoms, which, albeit different, could be fairly analog in pairs. More specifically, P(2) and P(3) are coordinated in a pseudo-trans position with each other, laying on a nearly straight line together with the Rh(2)-Rh(3) edge. Conversely, P(1) and P(4), separated by the Rh(1)-Rh(4) edge, form a narrower angle with each other. This relative spatial arrangement could highly affect the values of the coupling constant ^3^*J*_P-P_, respectively, making it either of similar magnitude to the ^1^*J*_Rh-P_, in which case a triplet would be observed, or neglectable, leading to just a doublet only due to the Rh-P coupling. As a result, P(2) and P(3) gave rise to two triplets (δ: 29.26 (t, *J* = 136.1 Hz), 23.98 (t, *J* = 128.8 Hz) ppm), while P(1) and P(4) generated two doublets (δ: 35.79 (d, *J*_Rh-P_ = 149.7 Hz), 13.98 (d, *J*_Rh-P_ = 126.3 Hz) ppm). The coupling values for ^1^*J*_Rh-P_ and ^3^*J*_P-P_ for atoms P(2) and P(3) could not be distinguished from each other.

**Figure 5:**
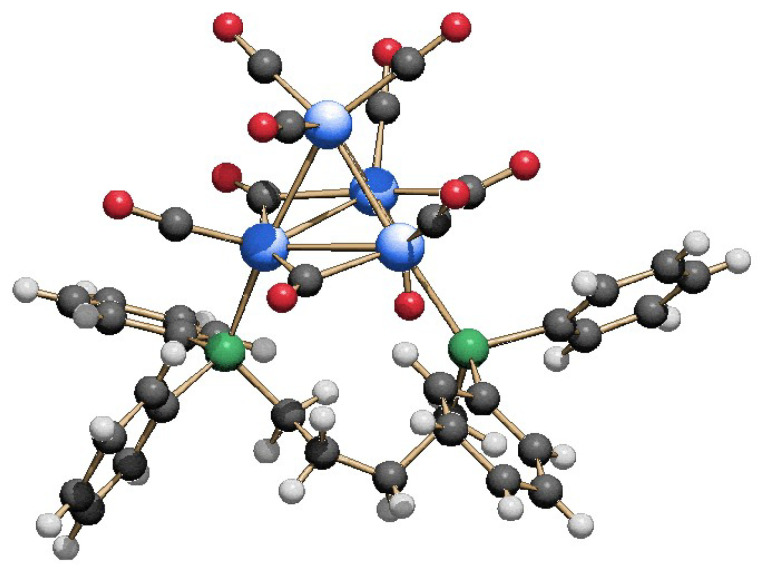
Structural model of Rh_4_(CO)_10_(dppb) (**3**) (blue, Rh; green, P; gray, C; red, O; white, H).

Compound **3** was obtained by adding one equivalent of dppb, in powder form, to a CH_2_Cl_2_ solution of the tetrarhodium carbonyl cluster precursor. At the end of the reaction, signaled by the downshift of the original ν_CO_ stretching values, the target compound was extracted in toluene, and its crystallization was achieved by layering hexane onto the solution. However, the low quality of the obtained crystals impaired a full disclosure of the crystal structure. Nonetheless, we were able to obtain a satisfactory structural model of Rh_4_(CO)_10_(dppb) (**3**), which is reported in Figure 5. In fact, the ligand arrangement exactly parallels that of compound **1**, both for the carbonyl and the bidentate phosphine.

**Figure 6:**
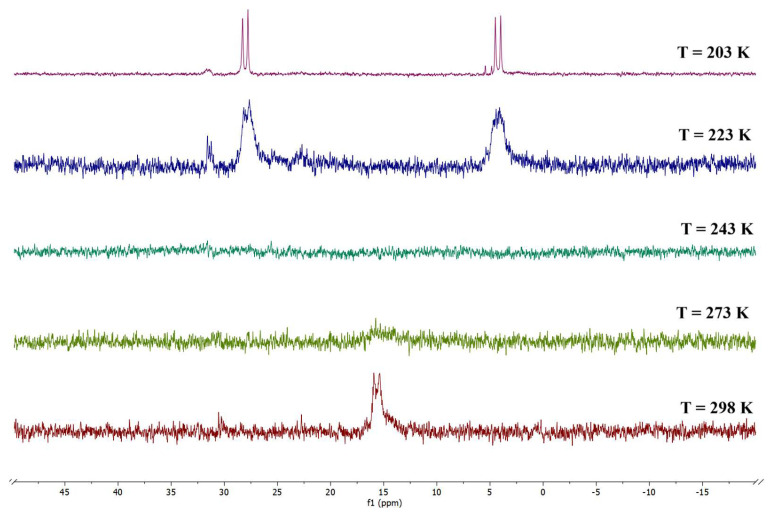
VT ^31^P{^1^H} NMR spectra of Rh_4_(CO)_10_(dppb) (**3**) in CD_2_Cl_2_ solution.

To deepen the characterization of **3**, a VT ^31^P{^1^H} NMR study was carried out. The spectra are reported in Figure 6. Similarly to what observed for cluster **1**, at room temperature, the phosphorus atoms of **3** were found chemically equivalent and they gave one single doublet (δ: 15.66 (d, *J*_Rh-P_ = 137.1 Hz) ppm). When the temperature decreased, the phosphorus signal coalesced, and no peaks were detectable until T = 223 K, where two clear doublets related to each phosphorus atom appeared (δ: 28.03 (d, *J*_Rh-P_ = 121.3 Hz), 4.24 (d, *J*_Rh-P_ = 123.2 Hz) ppm).

**Figure 7:**
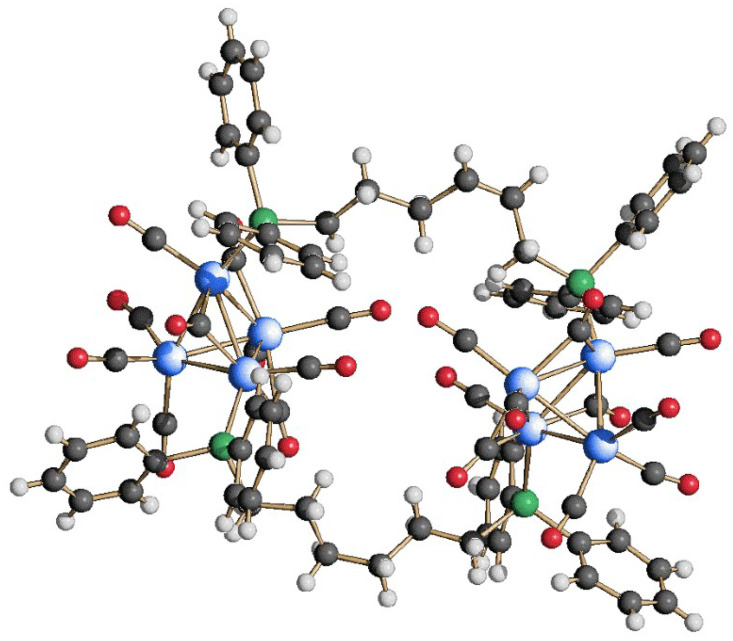
Molecular structure of {Rh_4_(CO)_10_(dpp-hexane)}_2_ (**4**) (blue, Rh; green, P; gray, C; red, O; white, H).

We expanded the reactivity of Rh_4_(CO)_12_ with a diphosphine bearing a longer alkyl carbon chain, namely dpp-hexane, and the results were quite different from those observed with dppe and dppb. More specifically, one equivalent of dpp-hexane was added to a CH_2_Cl_2_ solution of the Rh_4_(CO)_12_ cluster precursor, and after a few hours, the IR spectra showed a downshift of the ν_CO_ stretching frequencies, indicating the replacement of two carbonyl ligands by the phosphorus atoms of the diphosphine. However, the SC-XRD analysis performed on the obtained crystals showed the formation of the dimeric {Rh_4_(CO)_10_(dpp-hexane)}_2_ species (**4**). Its molecular structure, illustrated in Figure 7, consists of two Rh_4_(CO)_10_ tetrahedra linked through two molecules of dpp-hexane. Notably, each diphosphine acts as a monodentate ligand within the same cluster unit, as two rhodium atoms per cluster are coordinated to one phosphorus atom belonging to a different dpp-hexane. The ligand distribution has some similarities with that of compounds **1** and **3**. For instance, both metal vertexes have kept their three carbonyl ligands, so the phosphorus atoms are bonded to the triangular base in both cluster units. However, they are pointing away from each other and not in the same direction as in **1** and **3**, imparting a low grade of symmetry to the structure in the solid state.

**Figure 8:**
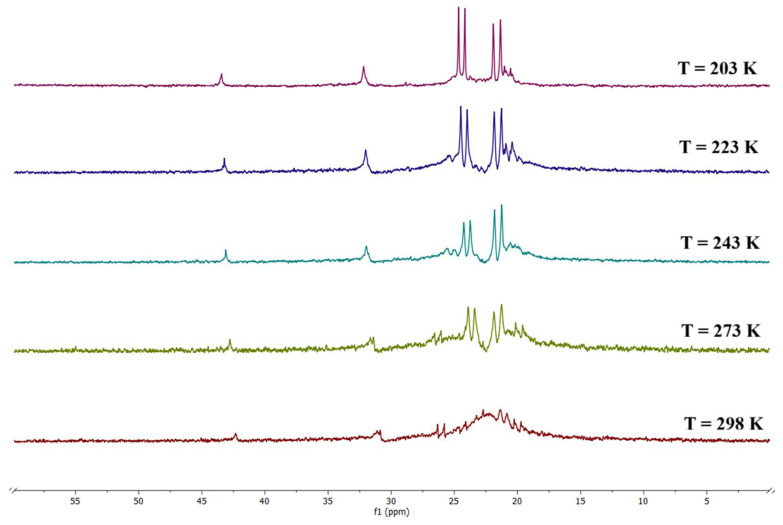
VT ^31^P{^1^H} NMR spectra of {Rh_4_(CO)_10_(dpp-hexane)}_2_ (**4**) in CD_2_Cl_2_ solution.

Indeed, at room temperature, the VT ^31^P{^1^H} NMR analysis, reported in Figure 8, showed that the phosphorus atoms are already coalescent. Conversely, at T = 203 K, two doublets appeared (δ: 24.42 (d, *J*_Rh-P_ = 120.0 Hz), 21.63 (d, *J*_Rh-P_ = 136.7 Hz) ppm), indicating that the phosphorus atoms belonging to the same diphosphine are chemically equivalent.

**Figure 9:**
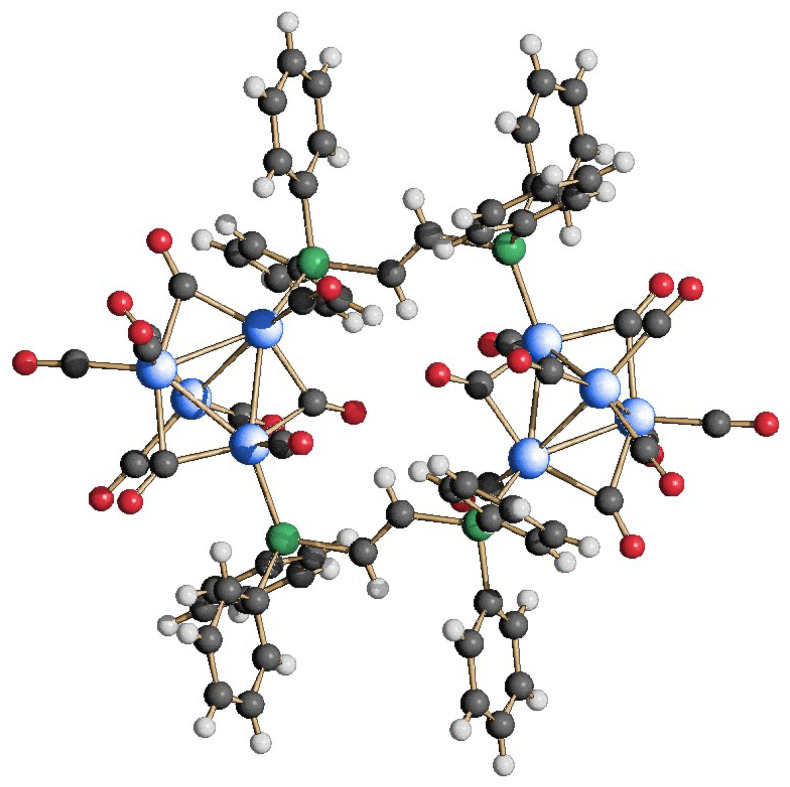
Molecular structure of {Rh_4_(CO)_10_(*trans*-dppe)}_2_ (**5**) (blue, Rh; green, P; gray, C; red, O; white, H).

A second dimeric species was obtained by reacting Rh_4_(CO)_12_ with one equivalent of *trans*-dppe, in the same previously described experimental conditions. Also in this case, the procedure was rather straightforward, and the reaction directly led to the dimeric {Rh_4_(CO)_10_(*trans*-dppe)}_2_ (**5**), which was structurally characterized by SC-XRD analysis. As shown in Figure 9, the two diphosphines are linking two Rh_4_(CO)_10_ tetrahedra in the same fashion seen for compound **4**, again acting as monodentate ligands within the same cluster unit. The similarity with **4** is also extended to the carbonyl coordination. However, the overall species possesses a center of symmetry, as the crystallographic asymmetric unit is composed of one Rh_4_(CO)_10_(*trans*-dppe) fragment.

**Figure 10:**
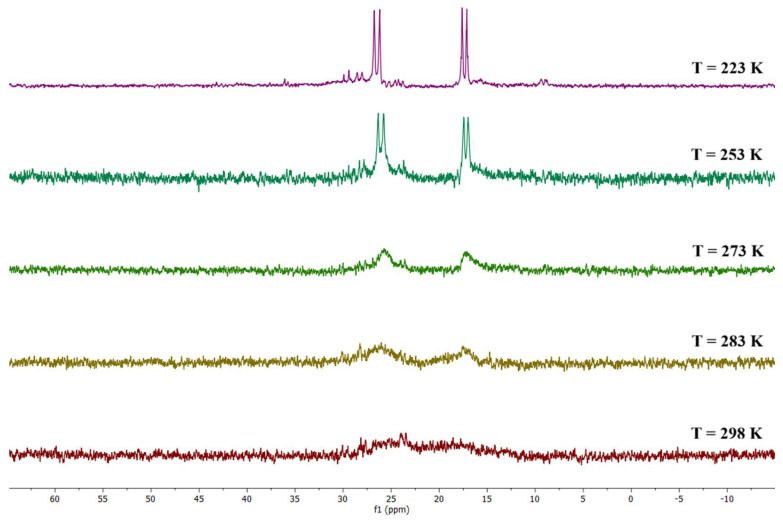
VT ^31^P{^1^H} NMR spectra of the species {Rh_4_(CO)_10_(*trans*-dppe)}_2_ in CD_2_Cl_2_ solution.

Due to such symmetry, the four phosphorus atoms are chemically equivalent in pairs. In fact, the ^31^P{^1^H} NMR spectrum recorded at T = 223 K showed two doublets (δ: 26.48 (d, *J*_Rh-P_ = 135.7 Hz), 17.38 (d, *J*_Rh-P_ = 123.2 Hz) ppm), corresponding to the phosphorus atoms in the same *trans*-dppe. Conversely, at room temperature, almost no signals were detectable, due to the coalescence of the phosphorus, which all become chemically equivalent (Figure 10).

Finally, we exploited the NMR spectra to determine the rate constants of the dynamic process occurring within the bidentate phosphine ligands through line shape simulation at different temperatures. More specifically, by means of the Eyring equation [31], it was possible to derive the activation energy Ea^‡^ and the enthalpy ΔH^‡^ of such dynamic processes at the coalescence temperature for compounds **1**, **3**, **4**, and **5**. The same analysis was not possible in the case of **2**, due to the high background noise present in the recorded ^31^P{^1^H} NMR spectra.

As reported in Table 1, the monomeric clusters **1** and **3** show similar values in terms of Ea^‡^ and ΔH^‡^, indicating a comparable energy barrier beyond which the phosphorus atoms become chemically equivalent. The difference between the coalescence temperatures is a consequence of the chemical shift. As a general trend, we can observe that the longer the alkyl chain between the two phosphorus atoms, the lower the Ea^‡^ and ΔH^‡^. This includes compound **4**. Compounds **1** and **3** also present a fairly similar ΔS^‡^, making their ΔG^‡^ values very close. The comparison between the dimeric species, conversely, reveals that their energy barriers are quite different, with **4** showing lower Ea^‡^ and ΔH^‡^ values than **5**. A possible reason could be due to the more rigid -CH=CH- unit in *trans*-dppe compared with the more flexible -C_6_H_12_- chain in dpp-hexane. Moreover, compound **4** presents the lowest ΔS^‡^, possibly due to an associative mechanism in the transition state, thus accounting for the highest ΔG^‡^ value.

## 3. Materials and Methods

**General procedures**

All reactions and sample manipulations were carried out at room temperature using standard Schlenk techniques under nitrogen and in dried solvents. All the reagents were commercial products (Aldrich, St. Louis, MO, USA) of the highest purity available and used as received, except for Rh_4_(CO)_12_, which has been prepared according to the literature [32]. IR spectra were recorded on a Perkin Elmer Spectrum One interferometer (Shelton, CT, USA) in CaF_2_ cells. ^31^P{^1^H} NMR measurements were performed on a Varian Inova 600 MHz instrument (Santa Clara, CA, USA), using an ATB probe. The phosphorus chemical shifts were referenced to external H_3_PO_4_ (85% in D_2_O). Elemental analyses were determined with a Thermo Quest Flash EA 1112NC instrument (Waltham, MA, USA). The diffraction experiments were carried out at room temperature on a Bruker APEX II diffractometer (Billerica, MA, USA) equipped with a PHOTON2 CMOS detector (PHOTONIS, Nemours, France), using Mo–Ka radiation. Data were corrected for Lorentz polarization and absorption effects (empirical absorption correction SADABS version 2.05) [33]. Structures were solved by direct methods and refined by full-matrix least-squares based on all data using F^2^ [34]. Hydrogen atoms were fixed at calculated positions and refined by a riding model. All non-hydrogen atoms were refined with anisotropic displacement parameters, including disordered atoms. In compound **2** and **4**, the SQUEEZE tool [35] was applied to address a problem of disordered solvent molecules. Anisotropic displacement parameter restraints were applied, when needed, to achieve a better structural model. Structure drawings were made with SCHAKAL99 [36].

**Synthesis of Rh_4_(CO)_10_(dppe) (1).**

A CH_2_Cl_2_ solution containing Rh_4_(CO)_12_ (0.180 g, 0.241 mmol) in 15 mL was reacted, under a nitrogen atmosphere, with dppe (0.096 g, 0.241 mmol) added as a powder. The solution was left under vigorous stirring for 6 h. The reaction crude was dried under a vacuum, and it was dissolved in toluene. The solution was layered with pentane, and crystals of Rh_4_(CO)_10_(dppe) were obtained (yield: 94.8% based on Rh). The compound is soluble in toluene, THF, and CH_2_Cl_2_; it is quite soluble in 2-propanol. The new cluster was characterized via IR spectroscopy, ^31^P{^1^H} NMR, and SC-XRD.

Rh_4_(CO)_10_(dppe). C_36_H_24_O_10_P_2_Rh_4_ (1090.15 g/mol): Calcd. (%): C 39.66, H 2.22; found (%): C 39.75, H 2.32. IR (toluene, 298 K) ν_CO_: 2068(s), 2040(s), 2015(vs), 1997(sh), 1881(w), 1844(m), 1809(m) cm^−1^. IR (Nujol, 298 K) ν_CO_: 2067(ms), 2031(s), 2004(ms), 2020(ms), 1992(ms), 1985(br), 1867(m), 1836(m), 1799(ms) cm^−1^. ^31^P{^1^H} NMR (CD_2_Cl_2_, 298 K) δ: 25.11 (d, *J*_Rh-P_ = 124.1 Hz) ppm. ^31^P{^1^H} NMR (CD_2_Cl_2_, 203 K) δ: 27.04 (d, *J*_Rh-P_ = 122.3 Hz), 23.53 (d, *J*_Rh-P_ = 123.6 Hz) ppm.

**Synthesis of Rh_4_(CO)_8_(dppe)_2_ (2).**

A CH_2_Cl_2_ solution containing Rh_4_(CO)_12_ (0.240 g, 0.321 mmol) in 15 mL was reacted, under a nitrogen atmosphere, with dppe (0.256 g, 0.642 mmol) added as a powder. The solution was left under vigorous stirring for 24 h. The reaction crude was dried under a vacuum, washed in toluene, and dissolved in THF. The solution was layered with hexane, and crystals of Rh_4_(CO)_8_(dppe)_2_ were obtained (yield: 19.8% based on Rh). The compound is soluble in THF and CH_2_Cl_2_. The new cluster was characterized via IR spectroscopy, ^31^P{^1^H} NMR, and SC-XRD.

Rh_4_(CO)_8_(dppe)_2_. C_60_H_48_O_8_P_4_Rh_4_ (1432.55 g/mol): Calcd. (%) C 50.31, H 3.38; found (%): C 50.42, H 3.43. IR (CH_2_Cl_2_, 298 K) ν_CO_: 2010(s), 1982(vs), 1971(sh), 1951(sh). 1805(m), 1783(m) cm^−1^. IR (Nujol, 298 K) ν_CO_: 2004(s), 1982(vs), 1964(vs), 1945(vs), 1867(m), 1802(m), 1771(s) cm^−1^. ^31^P{^1^H} NMR (CD_2_Cl_2_, 193 K) δ: 35.79 (d, *J*_Rh-P_ = 149.7 Hz), 29.26 (t, *J* = 136.1 Hz), 23.98 (t, *J* = 128.8 Hz), 13.98 (d, *J*_Rh-P_ = 126.3 Hz) ppm.

**Synthesis of Rh_4_(CO)_10_(dppb)·C_6_H_14_ (3·C_6_H_14_).**

A CH_2_Cl_2_ solution containing 0.200 g of Rh_4_(CO)_12_ (0.268 mmol) in 12 mL was reacted, under a nitrogen atmosphere, with 0.114 g of dppb (0.268 mmol) added as a powder. The solution was left under vigorous stirring for 6 h. The reaction crude was dried in a vacuum and extracted in toluene. The solution was layered with hexane, and crystals of Rh_4_(CO)_10_(dppb) C_6_H_14_ were obtained (yield: 87.4% based on Rh). The compound is soluble in THF, CH_2_Cl_2_, and toluene. The cluster was characterized via IR spectroscopy, ^31^P{^1^H} NMR, and SC-XRD.

Rh_4_(CO)_10_(dppb)·C_6_H_14_. C_44_H_42_O_10_P_2_Rh_4_ (1204.35 g/mol): Calcd. (%): C 43.89, H 3.32; found (%): C 44.15, H 3.44. IR (CH_2_Cl_2_, 298 K) ν_CO_: 2068(vs), 2039(s), 2014(vs), 1992(sh), 1882(w), 1847(m), 1813(m) cm^−1^. IR (Nujol, 298 K) ν_CO_: 2062(s), 2032(s), 2018(s), 1986(s), 1880(mw), 1848(m), 1806(m) cm^−1^. ^31^P{^1^H} NMR (CD_2_Cl_2_, 298 K) δ: 15.66 (d, *J*_Rh-P_ = 137.1 Hz) ppm. ^31^P{^1^H} NMR (CD_2_Cl_2_, 203 K) δ: 28.03 (d, *J*_Rh-P_ = 121.3 Hz), 4.24 (d, *J*_Rh-P_ = 123.2 Hz) ppm.

**Synthesis of {Rh_4_(CO)_10_(dpp** **-hexane)}_2_ (4).**

A CH_2_Cl_2_ solution containing Rh_4_(CO)_12_ (0.120 g, 0.160 mmol) in 15 mL was reacted, under a nitrogen atmosphere, with dpp-hexane (0.037 g, 0.080 mmol) added as a powder. The solution was left under vigorous stirring overnight. The reaction crude was dried under a vacuum and dissolved in toluene (10 mL). The solution was layered with hexane, and crystals of {Rh_4_(CO)_10_(dpp-hexane)}_2_ were obtained (yield: 90.8% based on Rh). The compound is soluble in toluene, THF, and CH_2_Cl_2_. The cluster was characterized via IR spectroscopy, ^31^P{^1^H} NMR, and X-ray diffractometry.

{Rh_4_(CO)_10_(dpp-hexane)}_2_. C_80_H_64_O_20_P_4_Rh_8_ (2292.51 g/mol): Calcd. (%): C 41.91, H 2.81; found (%): C 42.02, H 2.90. IR (CH_2_Cl_2_, 298 K) ν_CO_: 2064(s), 2039(vs), 2009(s), 1996(s), 1835(m), 1819(m) cm^−1^. IR (Nujol, 298 K) ν_CO_: 2084(m), 2053(sh), 2043(s), 2020(ms), 1867(m), 1834(m) cm^−1^. ^31^P{^1^H} NMR (CD_2_Cl_2_, 298 K) δ: 22.24 (br) ppm. ^31^P{^1^H} NMR (CD_2_Cl_2_, 203 K) δ: 24.42 (d, *J*_Rh-P_ = 120.0 Hz), 21.63 (d, *J*_Rh-P_ = 136.7 Hz) ppm.

**Synthesis of {Rh_4_(CO)_10_(*trans*-dppe)}_2_·2THF (5·2THF).**

A CH_2_Cl_2_ solution containing Rh_4_(CO)_12_ (0.200 g, 0.267 mmol) in 15 mL was reacted, under a nitrogen atmosphere, with *trans*-dppe (0.106 g, 0.267 mmol) added as a powder. The solution was left under vigorous stirring for six hours; then, the crude reaction was dried under a vacuum. Next, the solution was recovered in THF and layered with hexane to obtain crystals of {Rh_4_(CO)_10_(*trans*-dppe)}_2_·2THF (yield: 93.3% based on Rh). The compound is soluble in toluene, THF, and CH_2_Cl_2_; it is slightly soluble in acetonitrile. The new cluster was characterized via IR spectroscopy, ^31^P{^1^H} NMR, and SC-XRD.

{Rh_4_(CO)_10_(trans-dppe)}_2_·2THF. C_80_H_60_O_22_P_4_Rh_8_ (2320.48 g/mol): Calcd. (%): C 41.41, H 2.61; found (%): C 41.50, H 2.70. IR (CH_2_Cl_2_, 298 K) ν_CO_: 2068(s), 2047(vs), 2017(s), 1842(m), 1804(ms) cm^−1^. IR (Nujol, 298 K) ν_CO_: 2099(w), 2066(s), 2043(s), 1994(br), 1882(sh), 1837(s), 1791(s) cm^−1^. ^31^P{^1^H} NMR (CD_2_Cl_2_, 298 K) δ: 23.50 (br) ppm. ^31^P{^1^H} NMR (CD_2_Cl_2_, 223 K) δ: 26.48 (d, *J*_Rh-P_ = 135.7 Hz), 17.38 (d, *J*_Rh-P_ = 123.2 Hz) ppm.

## 4. Conclusions

In this paper, we have reported the synthesis and characterization of five novel diphosphine-substituted tetrarhodium heteroleptic carbonyl clusters, namely Rh_4_(CO)_10_(dppe) (**1**), Rh_4_(CO)_8_(dppe)_2_ (**2**), Rh_4_(CO)_10_(dppb) (**3**), {Rh_4_(CO)_10_(dpp-hexane)}_2_ (**4**), and {Rh_4_(CO)_10_(*trans*-dppe)}_2_ (**5**). Clusters **1**, **3**, **4**, and **5** were obtained in high yields by adding one equivalent of the corresponding diphosphine ligand in a 1:1 molar ratio with respect to the Rh_4_(CO)_12_ cluster precursor, in homogeneous conditions and under a nitrogen atmosphere. Notably, the alkyl chain length (ethane, butane, hexane) and the geometrical features of the aliphatic spacer (*trans*-ethylene) between the phosphorus atoms affected the outcome of the reactions. More specifically, when dppe and dppb were used, the diphosphines chelated two rhodium atoms on the same cluster, acting as intramolecular bidentate ligands, whereas dpp-hexane and *trans*-dppe chelated two rhodium atoms, each belonging to different cluster units, thus creating dimeric species.

In the case of dppe, which holds the shorter alkyl chain between the phosphorus atoms, it was also possible to coordinate a second bidentate ligand to the same cluster, yielding compound **2**. In the other cases, all various attempts failed to succeed; indeed, any further addition of the corresponding diphosphine resulted in the degradation of the cluster, which, in some cases, led to the formation of heteroleptic Rh_6_-based species, due to metal rearrangement. This phenomenon is known in the literature [20] and it is likely due to the steric hindrance of such ligands, also possibly in relation with the cluster size. In fact, in the case of the larger Rh_6_(CO)_16_ cluster, the bis-substituted Rh_6_(CO)_12_(dppb)_2_ species could be isolated. Another explanation for the unique obtainment of cluster **2** could be related to the experimental conditions. All reactions were carried out at room temperature, but it would be an interesting perspective to increase it and explore its role in the reaction paths.

All clusters were isolated and characterized via IR spectroscopy, ^31^P{^1^H} NMR studies at variable temperatures, and SC-XRD analysis. The IR spectra for all compounds showed an expected downshift of the ν_CO_ stretching frequencies with respect to those of the Rh_4_(CO)_12_ cluster precursor, due to an increased π-back-bonding interaction on the remaining carbonyl groups as a consequence of the lower Lewis acidity of the phosphine ligands. An even further downshift was observed in the case of cluster **2**, where two more carbonyl groups were replaced. The VT ^31^P{^1^H} NMR studies in solution totally confirmed the structural findings in the solid state related to the phosphorus coordination on the rhodium clusters. The crystal structures were determined through SC-XRD analyses at room temperature. In the case of compound **3**, the low diffraction power of the crystals prevented us from obtaining high-quality data; nonetheless, we were still able to obtain a satisfactory structural model that was completely in line with the IR and NMR findings.

Further studies will be devoted to enriching the library of diphosphine-substituted rhodium carbonyl cluster derivatives, and to testing their ability to act as catalysts in selected reactions. Furthermore, it would be interesting to investigate the possibility of isolating similar heteroleptic species but with larger-nuclearity rhodium carbonyl clusters, similar to what has been achieved, for instance, with platinum species [37].

## Figures and Tables

**Table 1:** Experimental values of the activation energy barriers for compounds **1**, **3**, **4** and **5**.

Cluster		Coalescence T (K)	ΔH^‡^ (kJ/mol)	Ea^‡^ (kJ/mol)	ΔS^‡^ (J/mol·K)	ΔG^‡^ (kJ/mol)
Rh_4_(CO)_10_(dppe)	(**1**)	243	44.42	46.45	12.10	41.60
Rh_4_(CO)_10_(dppb)	(**3**)	273	41.44	43.48	−4.124	42.61
{Rh_4_(CO)_10_(dpp-hexane)}_2_	(**4**)	298	11.60	13.63	−155.8	58.04
{Rh_4_(CO)_10_(*trans*-dppe)}_2_	(**5**)	298	35.37	33.23	35.60	24.75

## Data Availability

Deposition numbers 2512136–2512139 contain the supplementary crystallographic data for clusters **1**, **2**, **4,** and **5**, respectively. These data are provided free of charge by the joint Cambridge Crystallographic Data Centre and Fachinformationszentrum Karlsruhe Access Structures service.

## Supplementary Materials

The following supporting information can be downloaded at https://www.mdpi.com/article/10.3390/molecules31010193/s1. Figure S1. IR spectrum in the ν_CO_ region of Rh_4_(CO)_10_(dppe) (**1**) in CH_2_Cl_2_. Figure S2. IR spectrum in the ν_CO_ region of Rh_4_(CO)_10_(dppe) (**1**) in nujol mull. Figure S3. IR spectrum in the ν_CO_ region of Rh_4_(CO)_8_(dppe)_2_ (**2**) in CH_2_Cl_2_. Figure S4. IR spectrum in the ν_CO_ region of Rh_4_(CO)_8_(dppe)_2_ (**2**) in nujol mull. Figure S5. IR spectrum in the ν_CO_ region of Rh_4_(CO)_10_(dppb)·C_6_H_14_ (**3**·C_6_H_14_) in CH_2_Cl_2_. Figure S6. IR spectrum in the ν_CO_ region of Rh_4_(CO)_10_(dppb)·C_6_H_14_ (**3**·C_6_H_14_) in nujol mull. Figure S7. IR spectrum in the ν_CO_ region of {Rh_4_(CO)_10_(dpp-hexane)}_2_ (**4**) in CH_2_Cl_2_. Figure S8. IR spectrum in the ν_CO_ region of {Rh_4_(CO)_10_(dpp-hexane)}_2_ (**4**) in nujol mull. Figure S9. IR spectrum in the ν_CO_ region of {Rh_4_(CO)_10_(*trans*-dppe)}_2_·2THF (**5**·2THF) in CH_2_Cl_2_. Figure S10. IR spectrum in the ν_CO_ region of {Rh_4_(CO)_10_(*trans*-dppe)}_2_·2THF (**5**·2THF) in nujol mull. Figure S11. ^31^P{^1^H} NMR spectrum of Rh_4_(CO)_10_(dppe) (**1**) in CD_2_Cl_2_ at T = 298K. Figure S12. ^31^P{^1^H} NMR spectrum of Rh_4_(CO)_10_(dppe) (**1**) in CD_2_Cl_2_ at T = 203K. Figure S13. ^31^P{^1^H} NMR spectra at variable temperature of Rh_4_(CO)_8_(dppe)_2_ (**2**) in CD_2_Cl_2_. Figure S14. ^31^P{^1^H} NMR spectrum of Rh_4_(CO)_8_(dppe)_2_ (**2**) in CD_2_Cl_2_ at T = 298K. Figure S15. Magnification of the ^31^P{^1^H} NMR spectrum of Rh_4_(CO)_8_(dppe)_2_ (**2**) in CD_2_Cl_2_ at T = 193K. Figure S16. ^31^P{^1^H} NMR spectrum of Rh_4_(CO)_10_(dppb) (**3**) in CD_2_Cl_2_ at T = 298K. Figure S17. ^31^P{^1^H} NMR spectrum of Rh_4_(CO)_10_(dppb) (**3**) in CD_2_Cl_2_ at T = 203K. Figure S18. ^31^P{^1^H} NMR spectrum of {Rh_4_(CO)_10_(dpp-hexane)}_2_ (**4**) in CD_2_Cl_2_ at T = 298K. Figure S19. ^31^P{^1^H} NMR spectrum of {Rh_4_(CO)_10_(dpp-hexane)}_2_ (**4**) in CD_2_Cl_2_ at T = 203K. Figure S20. ^31^P{^1^H} NMR spectrum of {Rh_4_(CO)_10_(*trans*-dppe)}_2_ (**5**) in CD_2_Cl_2_ at T = 298K. Figure S21. ^31^P{^1^H} NMR spectrum of {Rh_4_(CO)_10_(*trans*-dppe)}_2_ (**5**) in CD_2_Cl_2_ at T = 223K. Figure S22. Metal skeleton and chelating atoms of Rh_4_(CO)_10_(dppe) (**1**). Figure S23. Metal skeleton and chelating atoms of Rh_4_(CO)_8_(dppe)_2_ (**2**). Figure S24. Metal skeleton and chelating atoms of {Rh_4_(CO)_10_(dpp-hexane)}_2_ (**4**). Figure S25. Metal skeleton and chelating atoms of {Rh_4_(CO)_10_(*trans*-dppe)}_2_ (**5**). Table S1. Crystallographic Table for **1**, **2**, **4** and **5**. Table S2. Selected bond lengths (Å) from the crystallographic analysis of **Rh_4_(CO)_10_(dppe) (1)**. Table S3. Selected bond lengths (Å) from the crystallographic analysis of **Rh_4_(CO)_8_(dppe)_2_ (2)**. Table S4. Selected bond lengths (Å) from the crystallographic analysis of **{Rh_4_(CO)_10_(dpp-hexane)}_2_** (**4**). Table S5. Selected bond lengths (Å) from the crystallographic analysis of **{Rh_4_(CO)_10_(*trans*-dppe)}_2_·2THF (5·2THF)**.
