# Supplementary material for: Diphosphine-Substituted Rhodium Carbonyl Clusters: Synthesis and Structural and Spectroscopic Characterization of the Heteroleptic Rh4(CO)8+2n(L)2−n (n = 0, 1) and {Rh4(CO)10L}2 Monomeric and Dimeric Species
Source: Molecules. 2026 Jan 5;31(1):193. doi: 10.3390/molecules31010193 (PMC12788074; doi:10.3390/molecules31010193)
Supplement: Supplementary file 1 [file molecules-31-00193-s001.zip › molecules-4046600-supplementary.pdf]

SUPPORTING INFORMATION FOR

Diphosphine-Substituted Rhodium Carbonyl Clusters: Synthesis and Structural and Spectroscopic Characterization of the Heteroleptic $Rh_4(CO)_{8+2n}(L)_{2-n}$ (n = 0, 1) and $\{Rh_4(CO)_{10}L\}_2$ Monomeric and Dimeric Species

Giorgia Scorzoni, Guido Bussoli, Cristiana Cesari, Maria Carmela Iapalucci *, Stefano Zacchini and Cristina Femoni *

Department of Industrial Chemistry "Toso Montanari", University of Bologna, Via Gobetti 85, 40129 Bologna, Italy; giorgia.scorzoni3@unibo.it (G.S.); bussoliguido@gmail.com (G.B.); cristiana.cesari2@unibo.it (C.C.); stefano.zacchini@unibo.it (S.Z.)

* Correspondence: cristina.femoni@unibo.it (C.F.); maria.iapalucci@unibo.it (M.C.I.)

Index

	Page/s
IR spectra	S2-S6
NMR spectra	S6-S10
X-ray crystallographic details	S13-S17

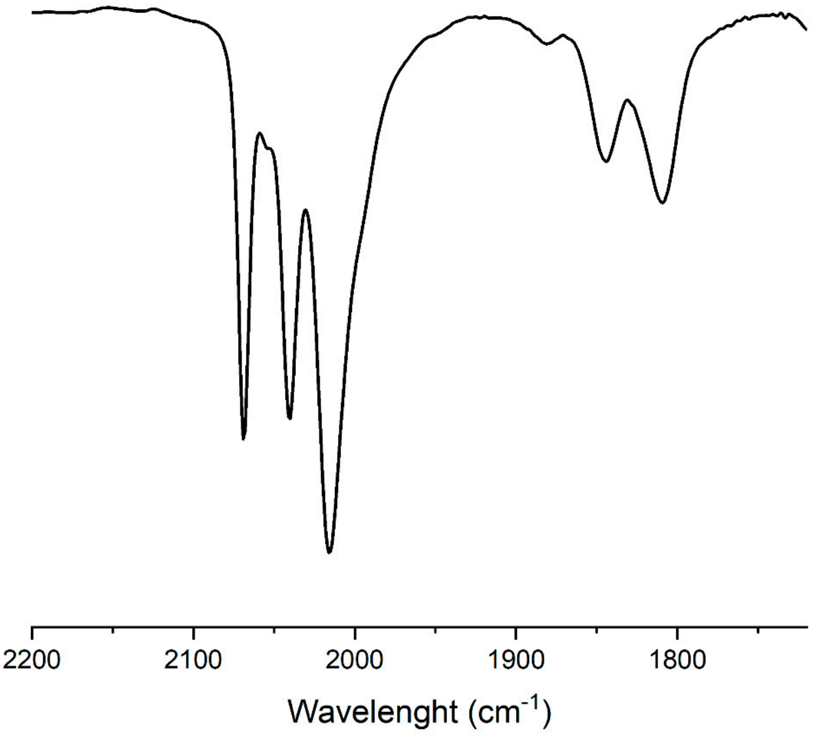

Figure S1. IR spectrum in the ν_{CO} region of $Rh_4(CO)_{10}$(dppe) (**1**) in CH_2Cl_2.

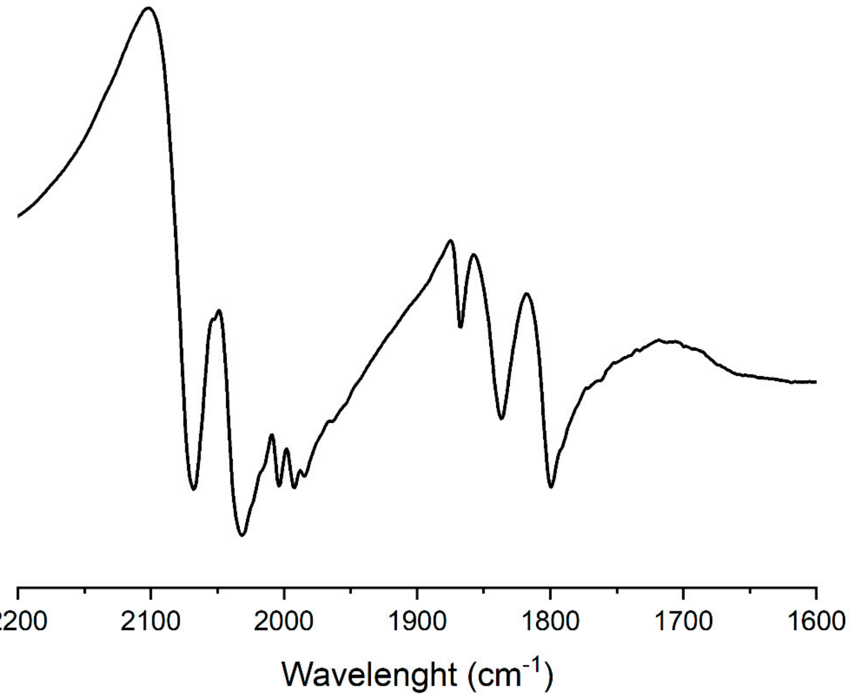

Figure S2. IR spectrum in the ν_{CO} region of $Rh_4(CO)_{10}$(dppe) (**1**) in nujol mull.

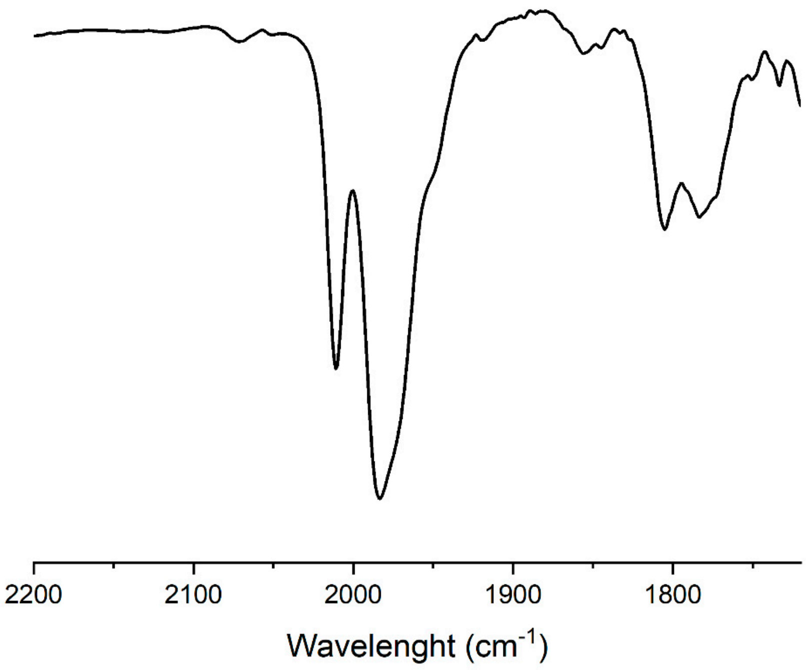

Figure S3. IR spectrum in the ν_{CO} region of $Rh_4(CO)_8(dppe)_2$ (**2**) in CH_2Cl_2.

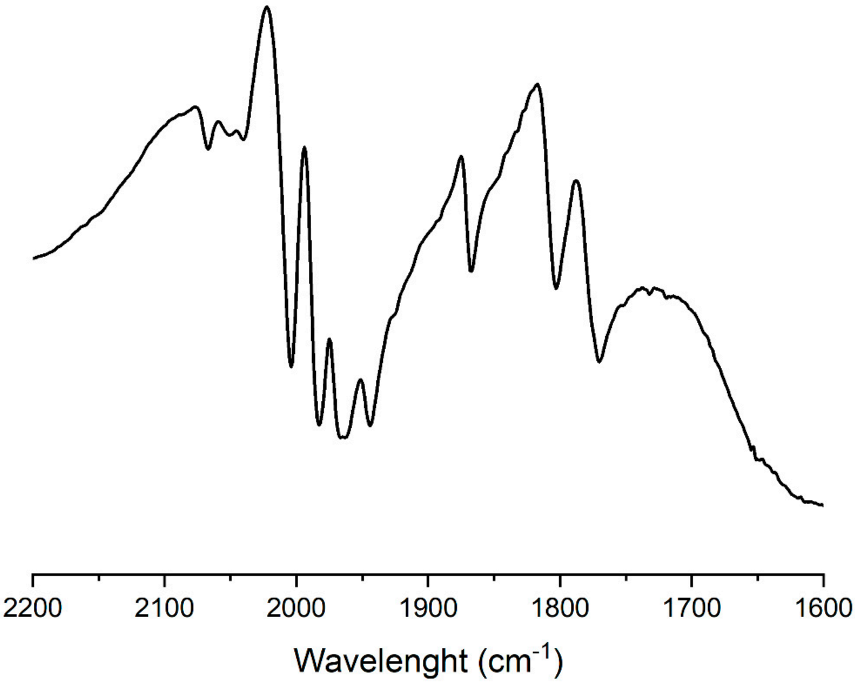

Figure S4. IR spectrum in the ν_{CO} region of $Rh_4(CO)_8(dppe)_2$ (**2**) in nujol mull.

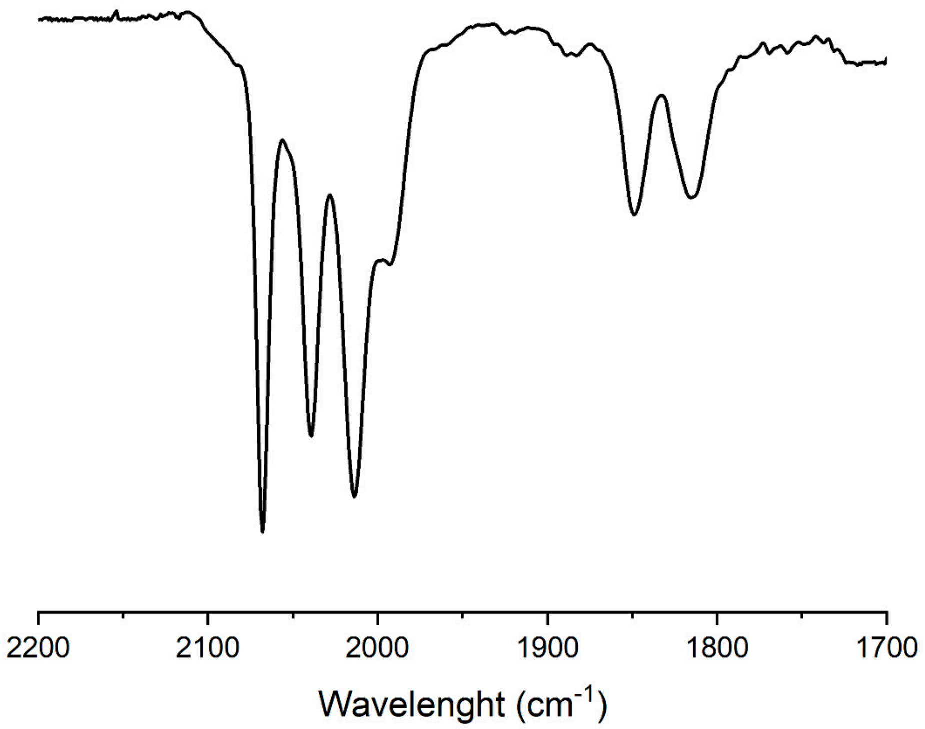

Figure S5. IR spectrum in the ν_{CO} region of $Rh_4(CO)_{10}(dppb)\cdot C_6H_{14}$ (**3**$\cdot C_6H_{14}$) in CH_2Cl_2.

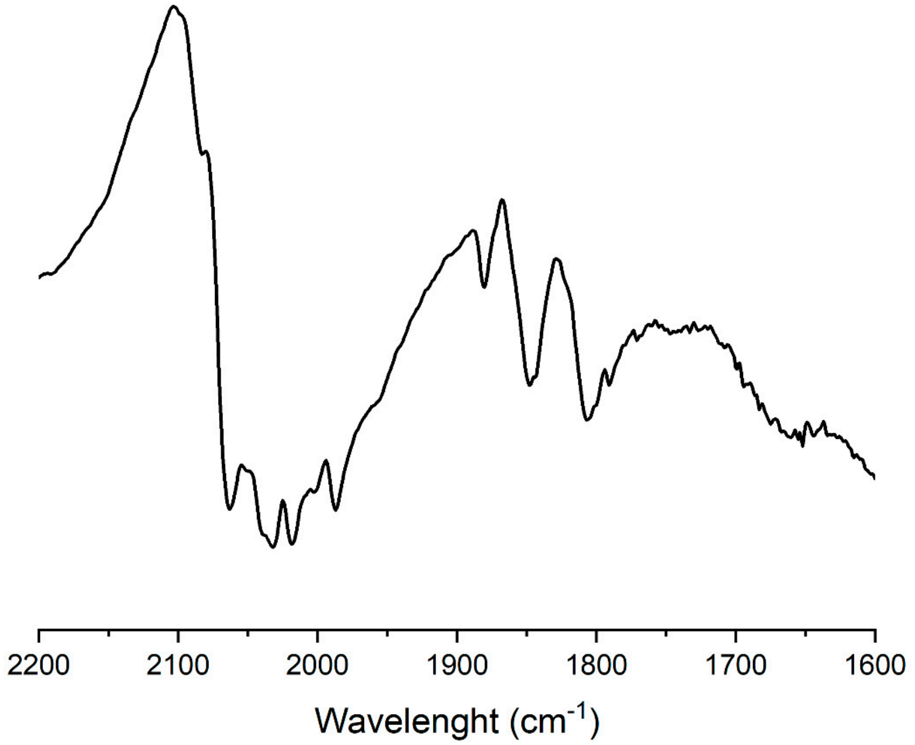

Figure S6. IR spectrum in the ν_{CO} region of $Rh_4(CO)_{10}(dppb)\cdot C_6H_{14}$ (**3**$\cdot C_6H_{14}$) in nujol mull.

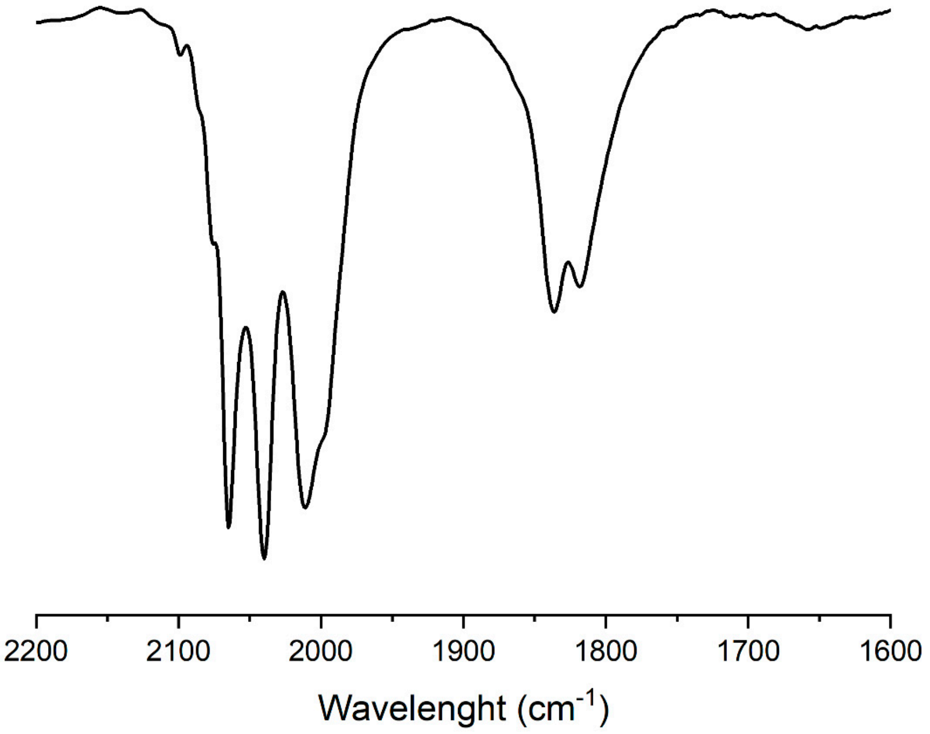

Figure S7. IR spectrum in the ν_{CO} region of $\{Rh_4(CO)_{10}(dpp\text{-}hexane)\}_2$ (**4**) in CH_2Cl_2.

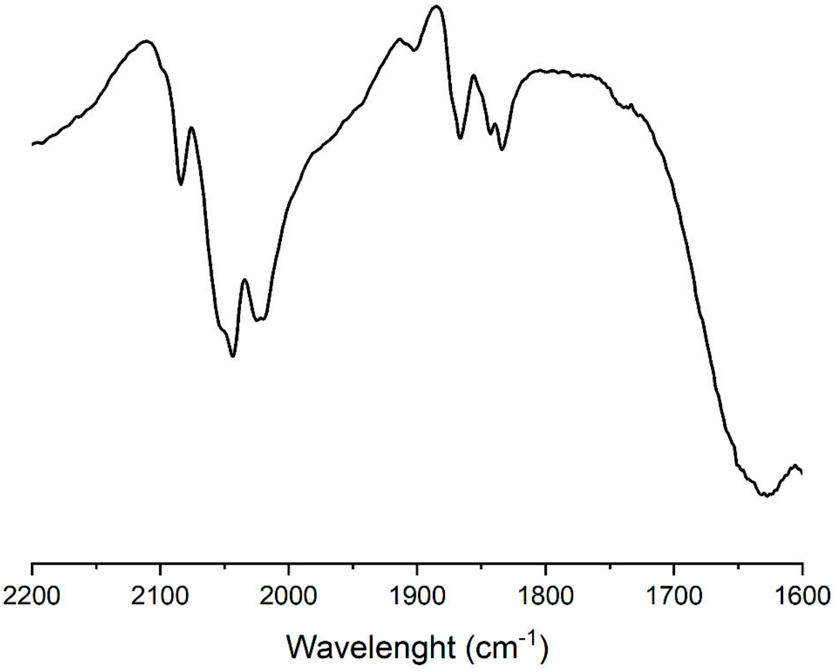

Figure S8. IR spectrum in the ν_{CO} region of $\{Rh_4(CO)_{10}(dpp\text{-}hexane)\}_2$ (**4**) in nujol mull.

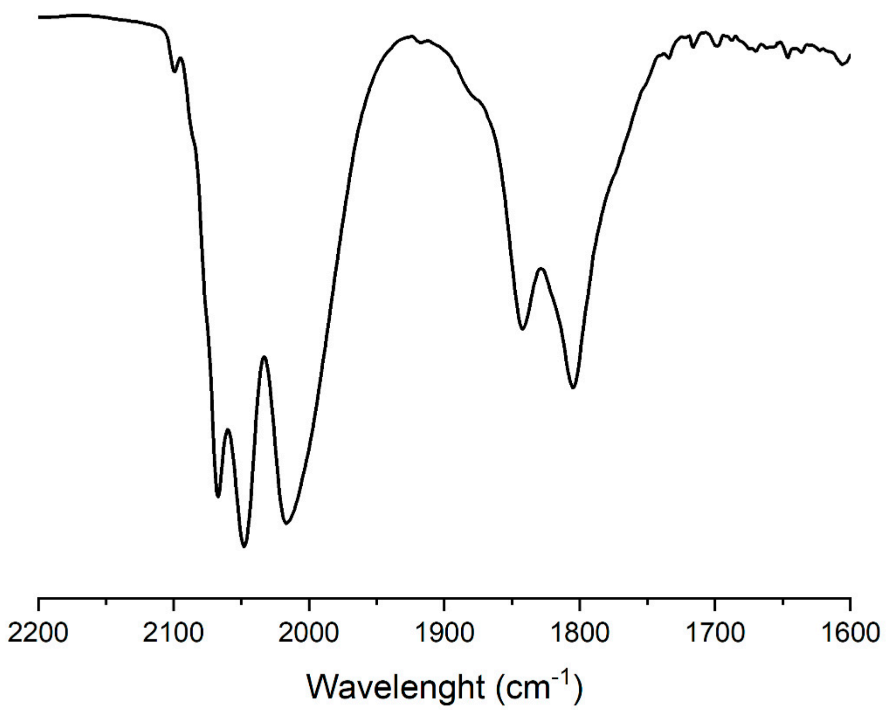

Figure S9. IR spectrum in the ν_{CO} region of $\{Rh_4(CO)_{10}($*trans*-dppe$)\}_2 \cdot 2THF$ (**5**·2THF) in CH_2Cl_2.

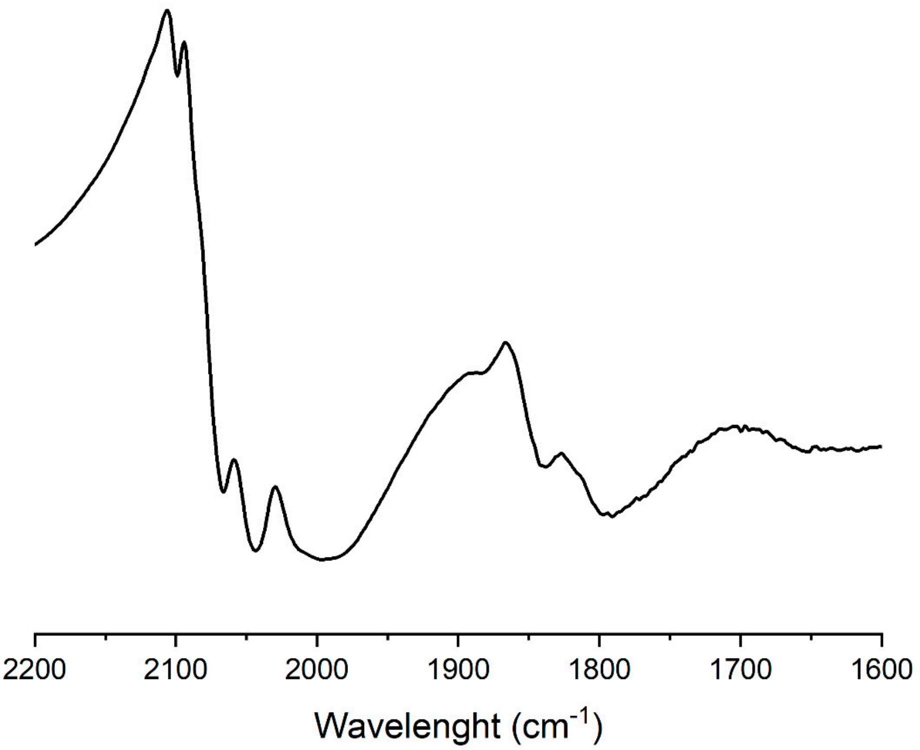

Figure S10. IR spectrum in the ν_{CO} region of $\{Rh_4(CO)_{10}($*trans*-dppe$)\}_2 \cdot 2THF$ (**5**·2THF) in nujol mull.

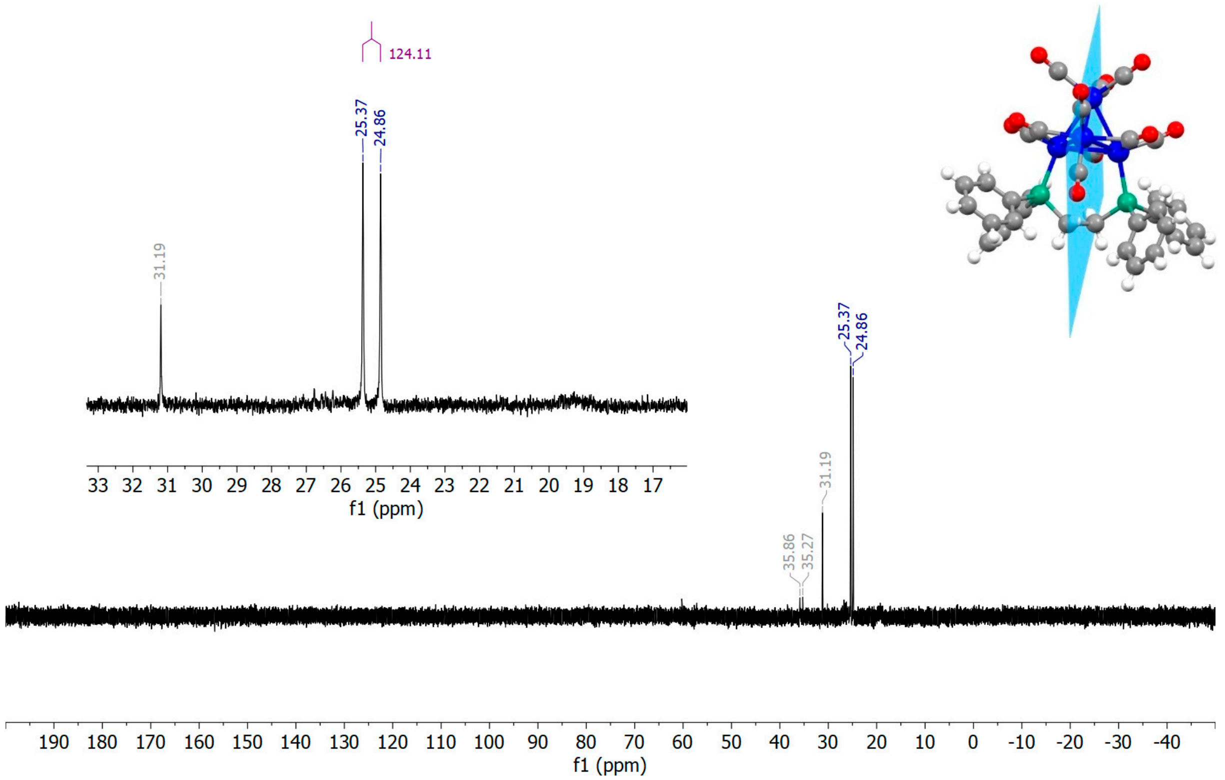

Figure S11. $^{31}P\{^{1}H\}$ NMR spectrum of $Rh_4(CO)_{10}(dppe)$ (**1**) in CD_2Cl_2 at T = 298K.

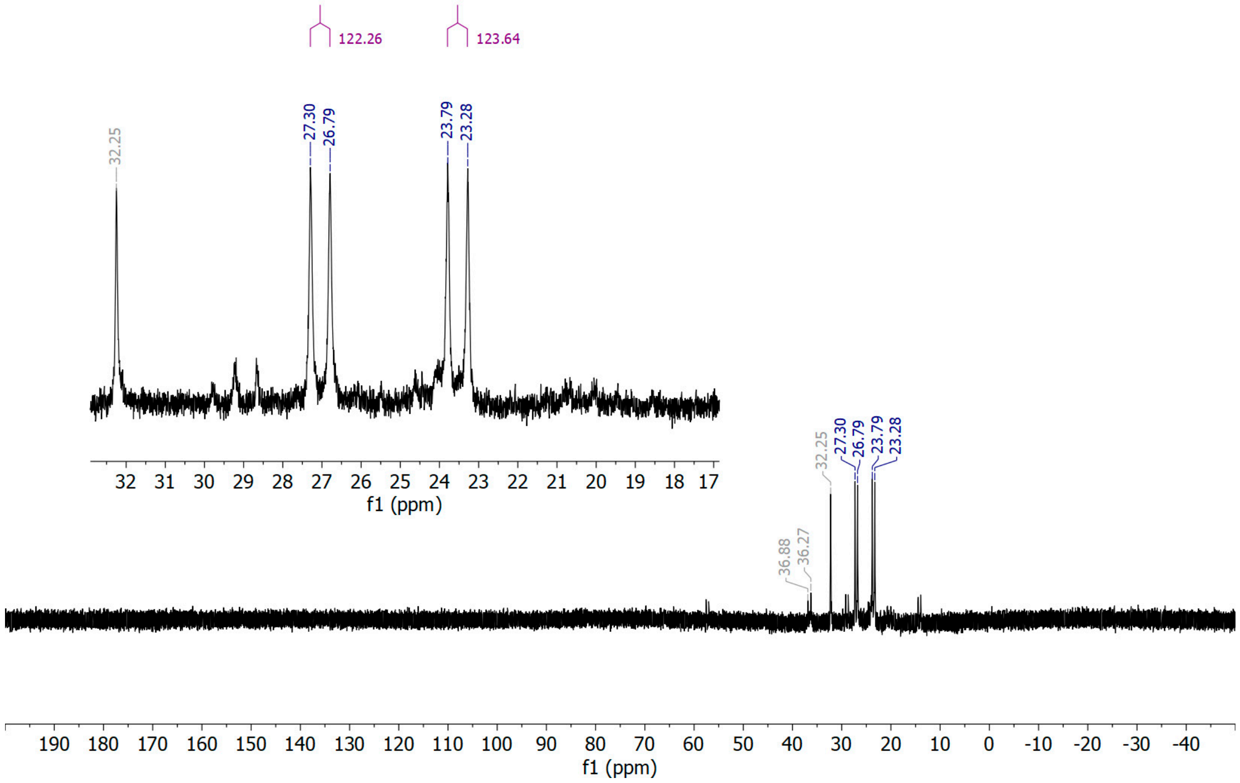

Figure S12. $^{31}P\{^{1}H\}$ NMR spectrum of $Rh_4(CO)_{10}(dppe)$ (**1**) in CD_2Cl_2 at T = 203K.

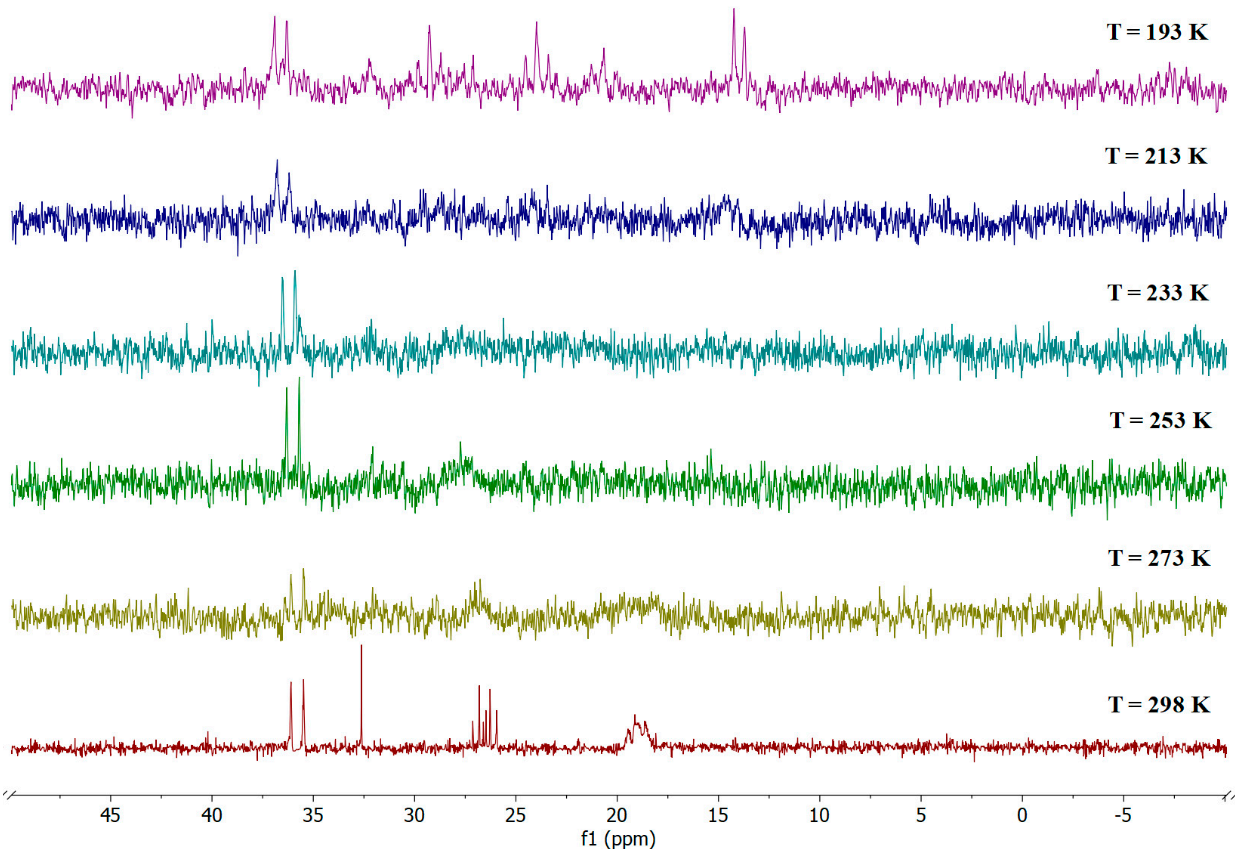

Figure S13. $^{31}P\{^1H\}$ NMR spectra at variable temperature of $Rh_4(CO)_8(dppe)_2$ (**2**) in CD_2Cl_2.

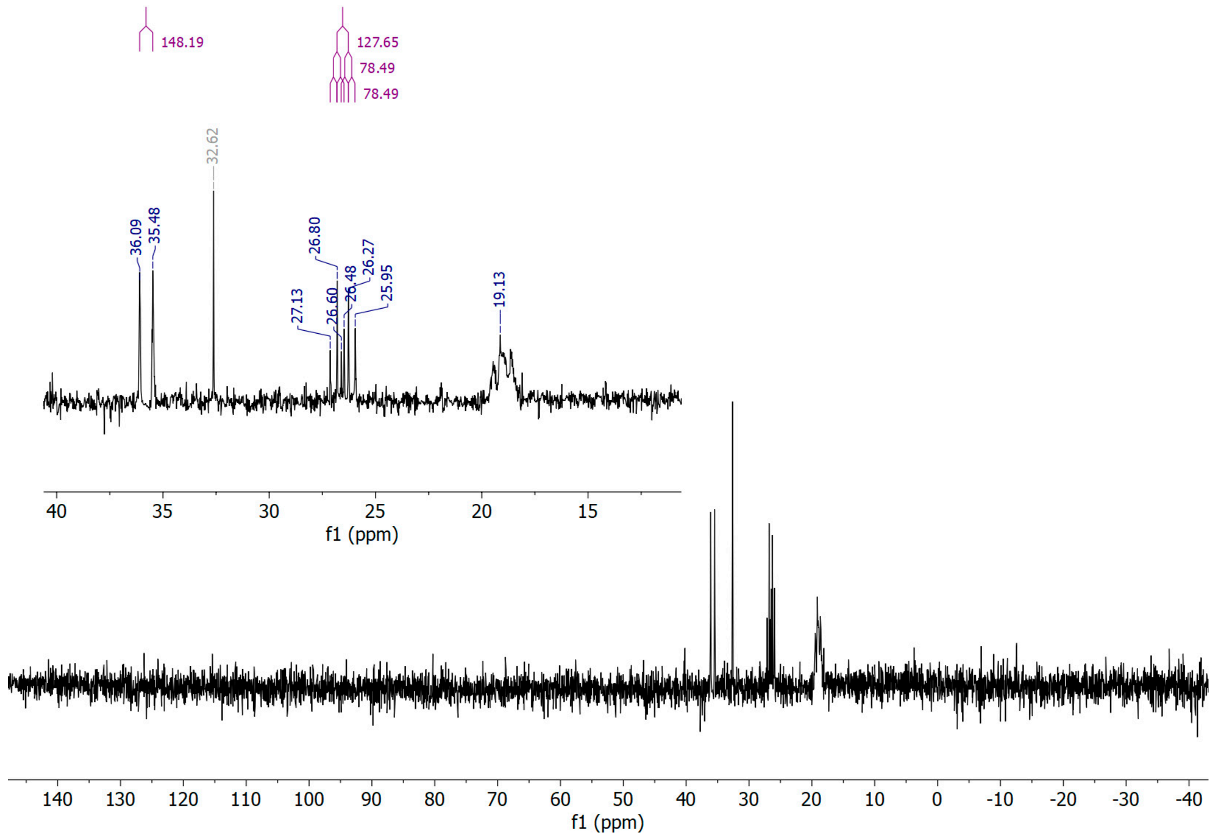

Figure S14. $^{31}P\{^1H\}$ NMR spectrum of $Rh_4(CO)_8(dppe)_2$ (**2**) in CD_2Cl_2 at T = 298K.

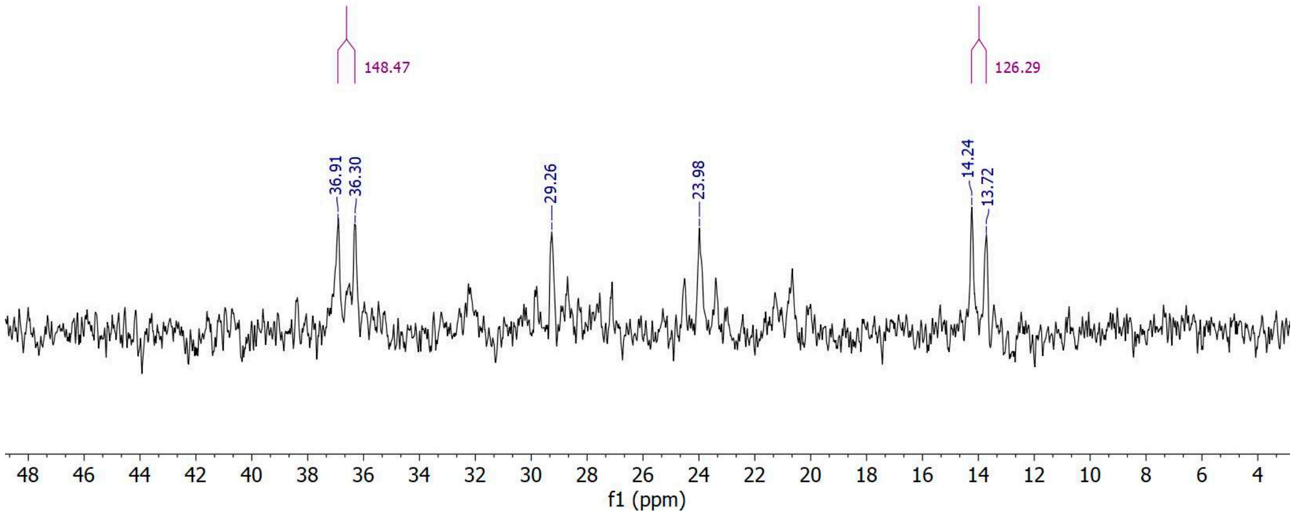

Figure S15. Magnification of the $^{31}P\{^1H\}$ NMR spectrum of $Rh_4(CO)_8(dppe)_2$ (**2**) in CD_2Cl_2 at T = 193K.

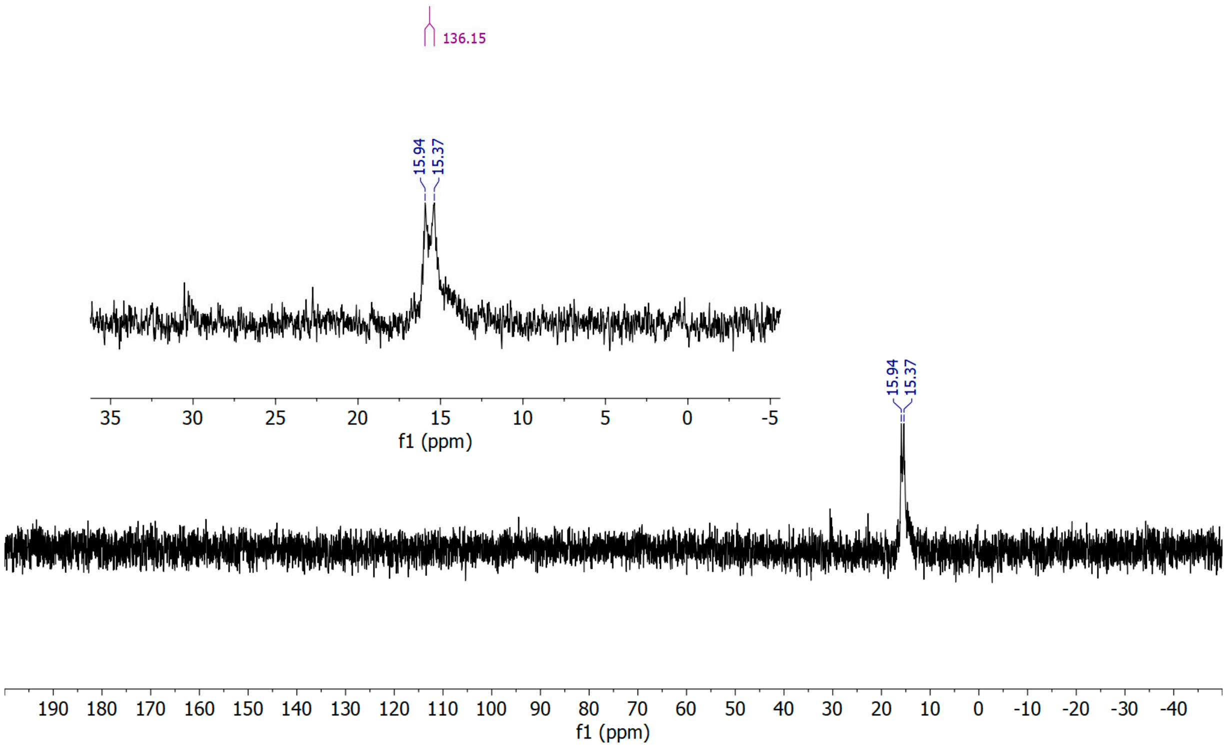

Figure S16. $^{31}P\{^1H\}$ NMR spectrum of $Rh_4(CO)_{10}(dppb)$ (**3**) in CD_2Cl_2 at T = 298K.

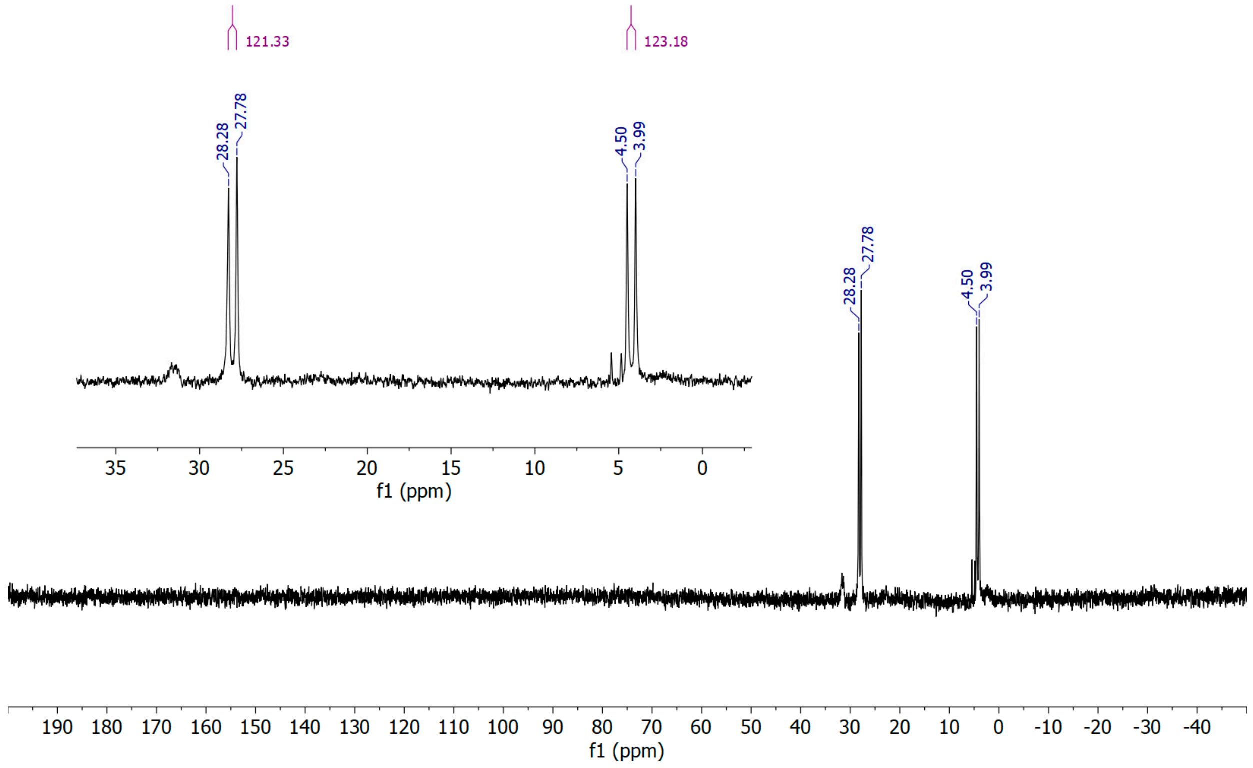

Figure S17. $^{31}P\{^1H\}$ NMR spectrum of $Rh_4(CO)_{10}$(dppb) (**3**) in CD_2Cl_2 at T = 203K.

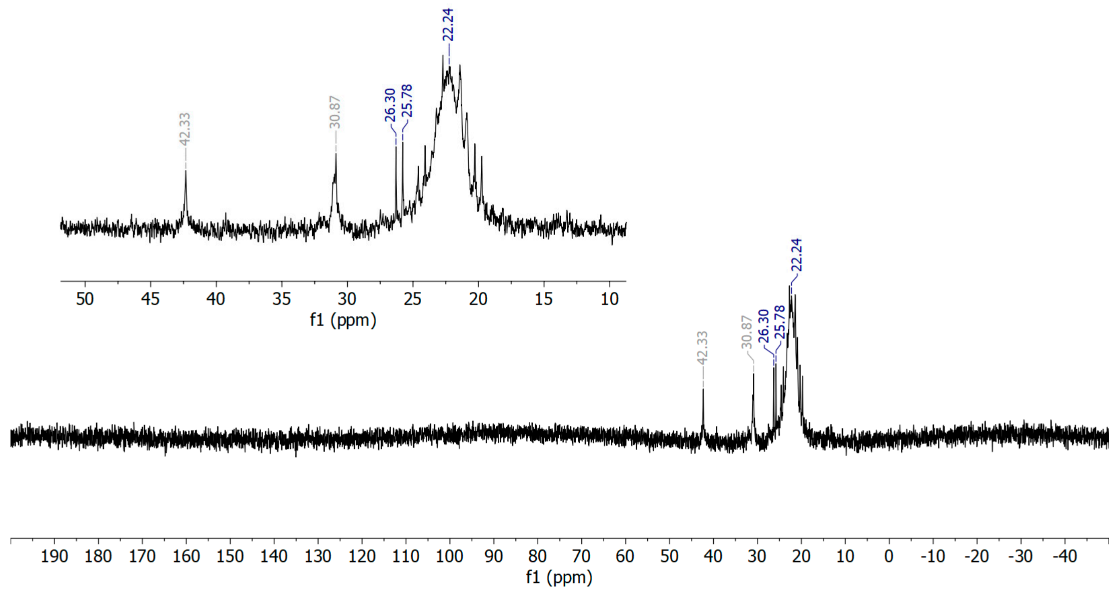

Figure S18. $^{31}P\{^1H\}$ NMR spectrum of $\{Rh_4(CO)_{10}$(dpp-hexane)$\}_2$ (**4**) in CD_2Cl_2 at T = 298K.

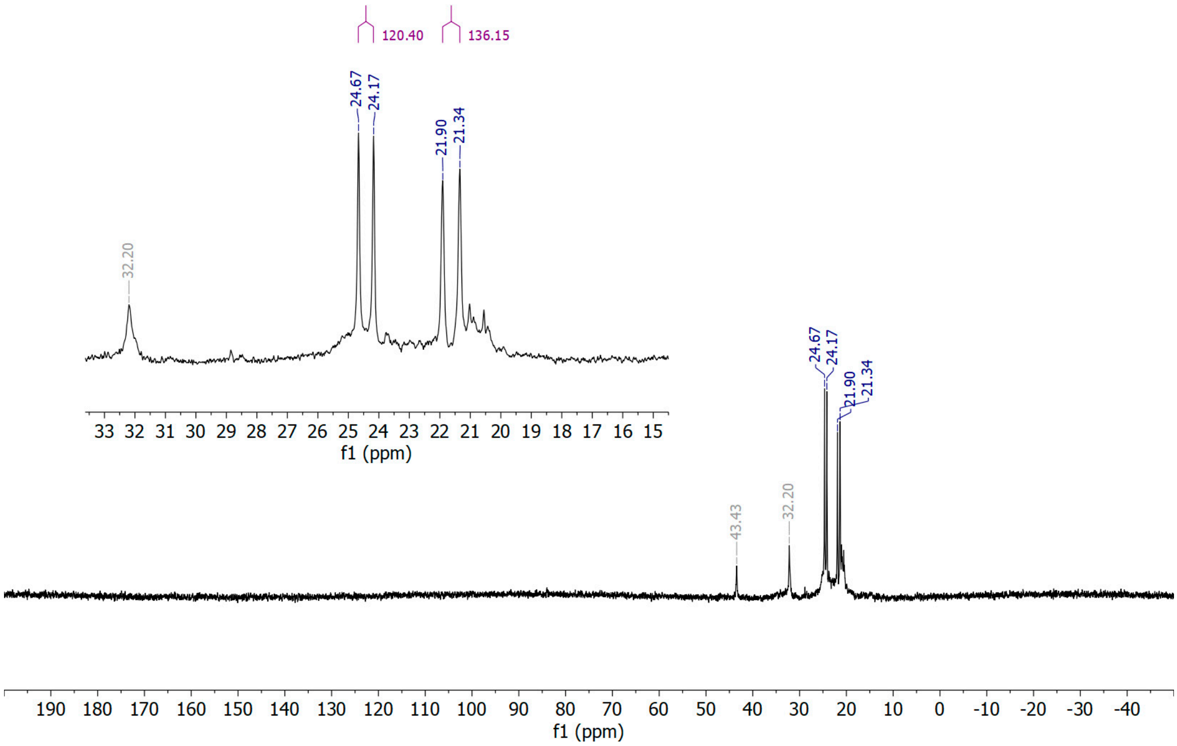

Figure S19. $^{31}P\{^{1}H\}$ NMR spectrum of $\{Rh_4(CO)_{10}$(dpp-hexane)$\}_2$ (**4**) in CD_2Cl_2 at T = 203K.

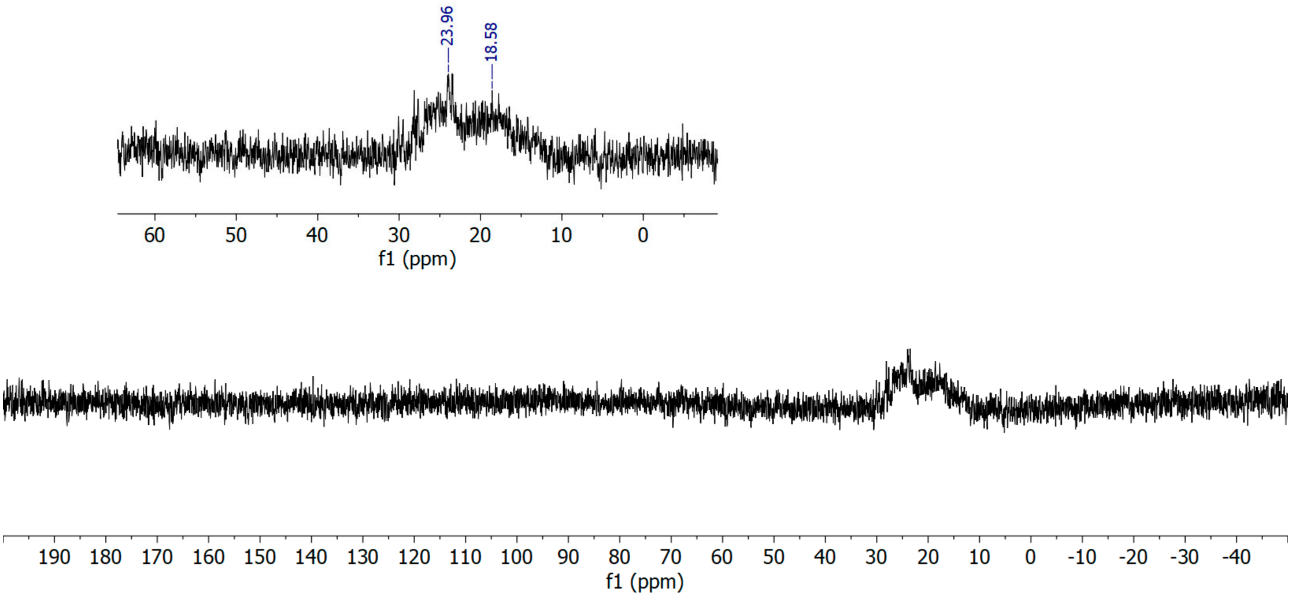

Figure S20. $^{31}P\{^{1}H\}$ NMR spectrum of $\{Rh_4(CO)_{10}$(*trans*-dppe)$\}_2$ (**5**) in CD_2Cl_2 at T = 298K.

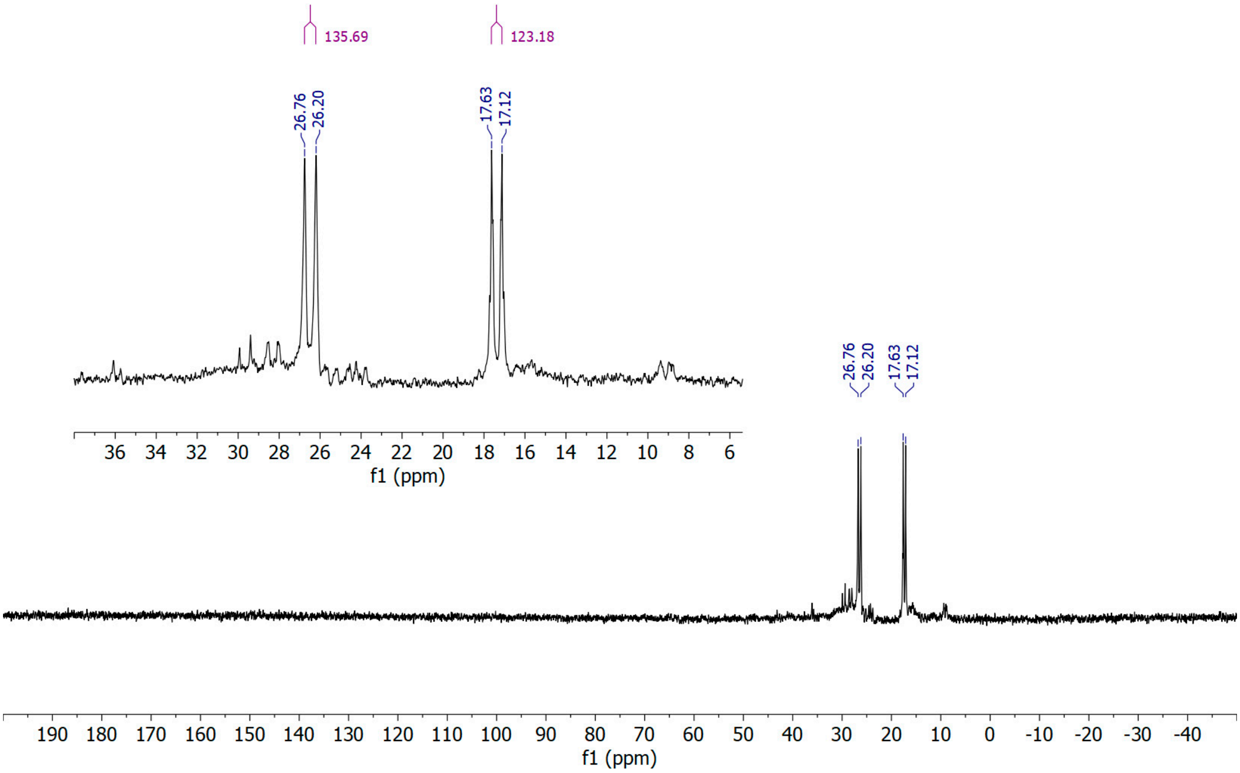

Figure S21. $^{31}P\{^1H\}$ NMR spectrum of $\{Rh_4(CO)_{10}($*trans*-dppe$)\}_2$ (**5**) in CD_2Cl_2 at T = 223K.

Table S1. Crystallographic Table for **1**, **2**, **4** and **5**.

	**$Rh_4(CO)_{10}(dppe)$ (1)**	**$Rh_4(CO)_8(dppe)_2$ (2)**	**$\{Rh_4(CO)_{10}(dpp\text{-}hexane)\}_2$ (4)**	**$\{Rh_4(CO)_{10}$(*trans*-dppe)$\}_2$•2THF (5)**
Formula	$C_{36}H_{24}O_{10}P_2Rh_4$	$C_{60}H_{48}O_8P_4Rh_4$	$C_{80}H_{64}O_{20}P_4Rh_8$	$C_{80}H_{60}O_{22}P_4Rh_8$
Fw	1090.13	1432.50	2292.47	2320.44
Crystal system	$P2_1/n$	$Pna2_1$	$P2_1/n$	C2/c
Space group	Monoclinic	Orthorhombic	Monoclinic	Monoclinic
a (Å)	15.8099(16)	23.0191(12)	19.4121(7)	30.484(5)
b (Å)	11.4407(13)	11.8437(6)	24.4064(9)	11.7089(15)
c (Å)	21.611(2)	23.1178(11)	25.4746(9)	26.483(3)
α (°)	90	90	90	90
β (°)	100.938(3)	90	112.3944(11)	108.741(8)
γ (°)	90	90	90	90
Cell volume ($Å^3$)	3837.9(7)	6302.6(5)	11159.1(7)	8951(2)
Z	4	4	4	4
D (g/cm^3)	1.887	1.510	1.365	1.722
μ (mm^{-1})	1.827	1.179	1.261	1.574
F(000)	2120	2848	4496	4544
θ limits (°)	2.022 to 24.998	2.125 to 25.000	1.669 to 25.000	1.776 to 24.500
Index ranges	-18<=h<=18, -13<=k<=13, -25<=l<=25	-27<=h<=27, -14<=k<=14, -27<=l<=27	-23<=h<=23, -29<=k<=29, -30<=l<=30	-35<=h<=35, -13<=k<=13, -30<=l<=30
Reflections collected	39065	80516	149017	55662
Independent reflections	6699 [R(int) = 0.0572]	11089 [R(int) = 0.2244]	19862 [R(int) = 0.0677]	7430 [R(int) = 0.1951]
Completeness to θ max	99.1%	99.8%	99.9%	99.9%
Data/restraints/parameters	6699 / 0 / 469	11089 / 385 / 638	19862 / 108 / 950	7430 / 222 / 548
Goodness of fit	1.126	1.048	1.059	1.072
R_1 (I > 2σ(I))	0.0467	0.0955	0.0895	0.1001
wR_2 (all data)	0.1129	0.2215	0.2516	0.2489
Largest diff. peak and hole, e $Å^{-3}$	0.773 and -1.273	1.926 and -1.106	1.662 and -1.539	1.134 and -1.776
Temperature	296(2) K	296(2) K	296(2) K	296(2) K

Table S2. Selected bond lengths (Å) from the crystallographic analysis of **$Rh_4(CO)_{10}$(dppe) (1)**.

Rh(1)-Rh(3)	2.6899(7)
Rh(1)-Rh(4)	2.7146(7)
Rh(1)-Rh(2)	2.7146(8)
Rh(2)-Rh(3)	2.6879(7)
Rh(2)-Rh(4)	2.7229(8)
Rh(3)-Rh(4)	2.6935(8)
Rh(1)-P(1)	2.2981(18)
Rh(2)-P(2)	2.3116(18)
Rh(1)-C(1)	1.878(8)
Rh(1)-C(3)	2.054(6)
Rh(1)-C(4)	2.063(7)
Rh(2)-C(2)	1.876(8)
Rh(2)-C(5)	2.049(8)
Rh(2)-C(3)	2.063(7)
Rh(3)-C(8)	1.926(9)
Rh(3)-C(9)	1.929(8)
Rh(3)-C(7)	1.940(9)
Rh(4)-C(10)	1.909(9)
Rh(4)-C(6)	1.917(11)

Rh(4)-C(4)	2.122(7)
Rh(4)-C(5)	2.124(8)
P(1)-C(11)	1.822(7)
P(1)-C(17)	1.827(7)
P(1)-C(51)	1.827(7)
P(2)-C(52)	1.824(7)
P(2)-C(31)	1.826(7)
P(2)-C(37)	1.832(7)
C(1)-O(1)	1.126(9)
C(2)-O(2)	1.124(9)
C(3)-O(3)	1.155(8)
C(4)-O(4)	1.147(8)
C(5)-O(5)	1.150(9)
C(6)-O(6)	1.110(12)
C(7)-O(7)	1.108(10)
C(8)-O(8)	1.121(10)
C(9)-O(9)	1.125(10)
C(10)-O(10)	1.127(10)

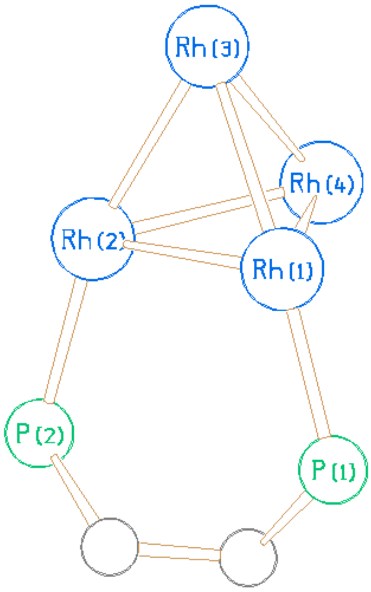

Figure S22. Metal skeleton and chelating atoms of $Rh_4(CO)_{10}$(dppe) (**1**).

Table S3. Selected bond lengths (Å) from the crystallographic analysis of **$Rh_4(CO)_8(dppe)_2$ (2)**.

Rh(1)-Rh(3)	2.736(3)
Rh(2)-Rh(1)	2.721(3)
Rh(2)-Rh(3)	2.679(2)
Rh(2)-Rh(4)	2.743(2)
Rh(4)-Rh(1)	2.712(3)
Rh(4)-Rh(3)	2.726(2)
Rh(1)-P(1)	2.299(7)
Rh(2)-P(2)	2.291(6)
Rh(3)-P(3)	2.329(6)
Rh(4)-P(4)	2.294(7)
Rh(1)-C(3)	1.85(3)
Rh(1)-C(6)	1.99(3)
Rh(1)-C(4)	2.09(2)
Rh(2)-C(5)	1.82(3)
Rh(2)-C(4)	2.02(3)
Rh(2)-C(2)	2.10(3)
Rh(3)-C(1)	1.89(3)
Rh(3)-C(8)	1.91(3)
Rh(4)-C(7)	1.83(3)
Rh(4)-C(6)	2.06(3)
Rh(4)-C(2)	2.11(3)

P(1)-C(100)	1.819(15)
P(1)-C(112)	1.83(2)
P(1)-C(106)	1.840(14)
P(2)-C(120)	1.83(3)
P(2)-C(114)	1.846(14)
P(2)-C(113)	1.87(3)
P(3)-C(212)	1.85(2)
P(3)-C(206)	1.85(3)
P(3)-C(200)	1.85(3)
P(4)-C(213)	1.78(2)
P(4)-C(214)	1.804(16)
P(4)-C(220)	1.81(3)
C(1)-O(1)	1.14(3)
C(2)-O(2)	1.16(3)
C(3)-O(3)	1.16(3)
C(4)-O(4)	1.21(3)
C(5)-O(5)	1.19(3)
C(6)-O(6)	1.22(3)
C(7)-O(7)	1.20(3)
C(8)-O(8)	1.14(4)

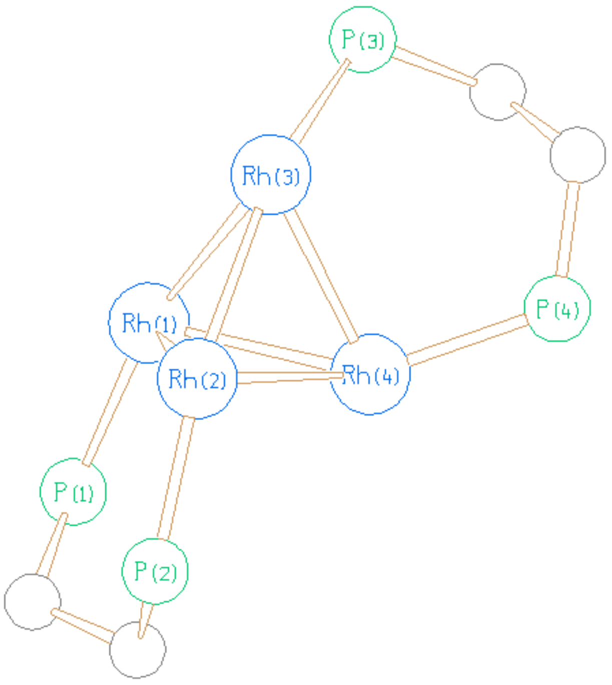

Figure S23. Metal skeleton and chelating atoms of $Rh_4(CO)_8(dppe)_2$ (**2**).

Table S4. Selected bond lengths (Å) from the crystallographic analysis of **{$Rh_4(CO)_{10}$(dpp-hexane)}$_2$** (**4**).

Rh(1)-Rh(4)	2.6897(14)	C(5)-O(5)	1.16(3)	Rh(12)-C(21)	2.098(13)
Rh(1)-Rh(2)	2.7189(13)	C(6)-O(6)	1.13(2)	Rh(13)-C(26)	1.86(2)
Rh(1)-Rh(3)	2.7391(15)	C(7)-O(7)	1.142(19)	Rh(13)-C(27)	1.87(2)
Rh(2)-Rh(3)	2.6889(14)	C(8)-O(8)	1.14(2)	Rh(13)-C(28)	1.937(19)
Rh(2)-Rh(4)	2.7683(14)	C(9)-O(9)	1.14(3)	Rh(14)-C(29)	1.93(2)
Rh(3)-Rh(4)	2.6861(16)	C(10)-O(10)	1.19(2)	Rh(14)-C(30)	1.98(3)
Rh(1)-P(1)	2.336(4)	P(1)-C(112)	1.803(15)	Rh(14)-C(22)	2.150(18)
Rh(2)-P(2)	2.319(4)	P(1)-C(100)	1.810(16)	Rh(14)-C(23)	2.208(19)
Rh(1)-C(4)	1.874(13)	P(1)-C(106)	1.855(17)	C(21)-O(21)	1.160(18)
Rh(1)-C(7)	2.044(15)	P(2)-C(132)	1.782(17)	C(22)-O(22)	1.143(19)
Rh(1)-C(3)	2.090(13)	P(2)-C(120)	1.825(9)	C(23)-O(23)	1.06(2)
Rh(2)-C(1)	1.896(13)	P(2)-C(126)	1.829(10)	C(24)-O(24)	1.053(19)
Rh(2)-C(2)	2.091(16)	Rh(11)-Rh(14)	2.6969(15)	C(25)-O(25)	1.13(2)
Rh(2)-C(3)	2.105(13)	Rh(11)-Rh(13)	2.7209(16)	C(26)-O(26)	1.20(2)
Rh(3)-C(10)	1.932(17)	Rh(11)-Rh(12)	2.7379(14)	C(27)-O(27)	1.26(3)
Rh(3)-C(6)	1.93(2)	Rh(12)-Rh(13)	2.6935(16)	C(28)-O(28)	1.13(3)
Rh(3)-C(8)	1.97(2)	Rh(12)-Rh(14)	2.7621(18)	C(29)-O(29)	1.05(2)
Rh(4)-C(9)	1.89(2)	Rh(13)-Rh(14)	2.680(2)	C(30)-O(30)	1.14(3)
Rh(4)-C(5)	1.91(3)	Rh(11)-P(11)	2.314(3)	P(11)-C(117)	1.831(15)
Rh(4)-C(7)	2.103(16)	Rh(12)-P(12)	2.313(4)	P(11)-C(200)	1.838(13)
Rh(4)-C(2)	2.113(15)	Rh(11)-C(24)	1.903(14)	P(11)-C(206)	1.849(8)
C(1)-O(1)	1.114(17)	Rh(11)-C(21)	2.104(16)	P(12)-C(137)	1.822(14)
C(2)-O(2)	1.170(19)	Rh(11)-C(23)	2.13(2)	P(12)-C(212)	1.815(8)
C(3)-O(3)	1.159(17)	Rh(12)-C(25)	1.866(14)	P(12)-C(218)	1.834(9)
C(4)-O(4)	1.06(2)	Rh(12)-C(22)	2.057(16)		

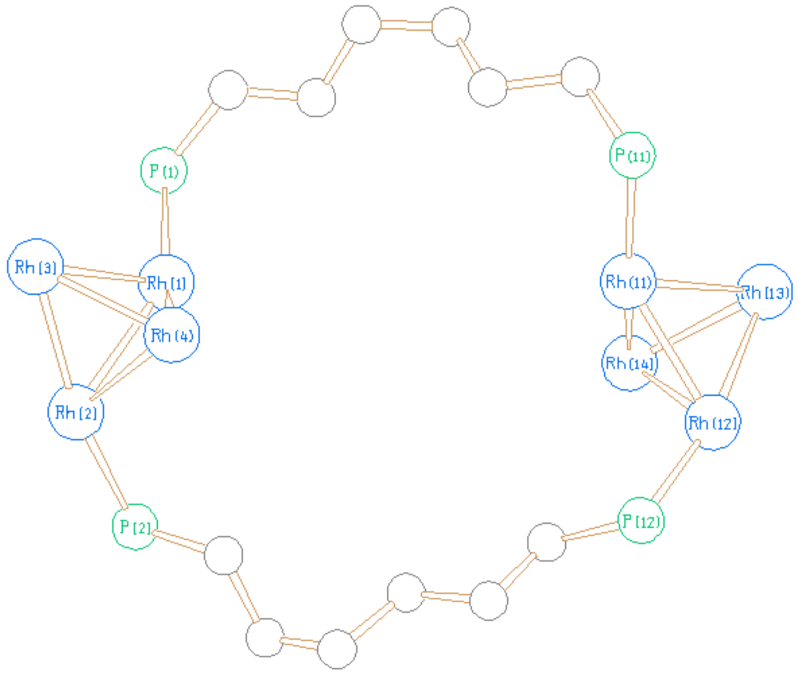

Figure S24. Metal skeleton and chelating atoms of {$Rh_4(CO)_{10}$(dpp-hexane)}$_2$ (**4**).

Table S5. Selected bond lengths (Å) from the crystallographic analysis of **{Rh$_4$(CO)$_{10}$(*trans*-dppe)}$_2$**·2THF (**5**·2THF).

Rh(1)-Rh(2)	2.7009(16)
Rh(1)-Rh(4)	2.7261(17)
Rh(1)-Rh(3)	2.7303(18)
Rh(2)-Rh(3)	2.7287(18)
Rh(2)-Rh(4)	2.7296(17)
Rh(3)-Rh(4)	2.6688(19)
Rh(1)-P(2)	2.319(4)
Rh(2)-P(1)	2.309(4)
Rh(1)-C(1)	2.041(17)
Rh(1)-C(2)	1.902(19)
Rh(1)-C(8)	2.067(13)
Rh(2)-C(6)	1.87(2)
Rh(2)-C(5)	2.05(2)
Rh(2)-C(8)	2.081(15)
Rh(3)-C(9)	1.89(2)
Rh(3)-C(7)	1.901(19)
Rh(3)-C(10)	1.96(2)
Rh(4)-C(3)	1.883(19)
Rh(4)-C(4)	1.91(3)

Rh(4)-C(5)	2.094(18)
Rh(4)-C(1)	2.150(19)
C(1)-O(1)	1.17(2)
C(2)-O(2)	1.13(2)
C(3)-O(3)	1.15(2)
C(4)-O(4)	1.13(3)
C(5)-O(5)	1.19(2)
C(6)-O(6)	1.15(2)
C(7)-O(7)	1.15(2)
C(8)-O(8)	1.184(16)
C(9)-O(9)	1.14(2)
C(10)-O(10)	1.12(2)
P(1)-C(100)	1.840(17)
P(1)-C(106)	1.809(17)
P(1)-C(112)	1.802(15)
P(2)-C(113)	1.847(17)
P(2)-C(206)	1.792(11)
P(2)-C(200)	1.828(16)

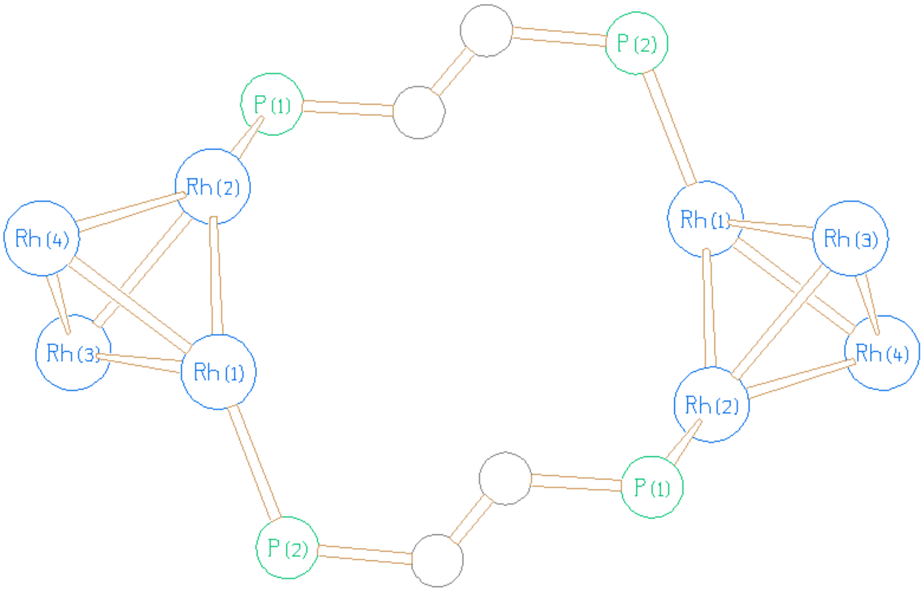

Figure S25. Metal skeleton and chelating atoms of {Rh$_4$(CO)$_{10}$(*trans*-dppe)}$_2$ (**5**).
